# Control of chicken CR1 retrotransposons is independent of Dicer-mediated RNA interference pathway

**DOI:** 10.1186/1741-7007-7-53

**Published:** 2009-08-19

**Authors:** Sung-Hun Lee, Preethi Eldi, Soo-Young Cho, Danny Rangasamy

**Affiliations:** 1 The John Curtin School of Medical Research, Australian National University, Canberra, Australian Capital Territory 2601, Australia; 2 Division of Molecular and Life Sciences, Hanyang University, Ansan, Kyunggi-do, Republic of Korea

## Abstract

**Background:**

Dicer is an RNase III-ribonuclease that initiates the formation of small interfering RNAs as a defence against genomic parasites such as retrotransposons. Despite intensive characterization in mammalian species, the biological functions of Dicer in controlling retrotransposable elements of the non-mammalian vertebrate are poorly understood. In this report, we examine the role of chicken Dicer in controlling the activity of chicken CR1 retrotransposable elements in a chicken-human hybrid DT40 cell line employing a conditional loss-of-Dicer function.

**Results:**

Retrotransposition is detrimental to host genome stability and thus eukaryotic cells have developed mechanisms to limit the expansion of retrotransposons by Dicer-mediated RNAi silencing pathways. However, the mechanisms that control the activity and copy numbers of transposable elements in chicken remain unclear. Here, we describe how the loss of Dicer in chicken cells does not reactivate endogenous chicken CR1 retrotransposons with impaired RNAi machinery, suggesting that the control of chicken CR1 is independent of Dicer-induced RNAi silencing. In contrast, upon introduction of a functionally active human L1 retrotransposable element that contains an active 5' UTR promoter, the Dicer-deficient chicken cells show a strong increase in the accumulation of human L1 transcripts and retrotransposition activity, highlighting a major difference between chicken CR1 and other mammalian L1 retrotransposons.

**Conclusion:**

Our data provide evidence that chicken CR1 retrotransposons, unlike their mammalian L1 counterparts, do not undergo retrotransposition because most CR1 retrotransposons are truncated or mutated at their 5'UTR promoters and thus are not subjected to Dicer-mediated RNAi-silencing control.

## Background

The chicken is an important model organism for the large-scale analysis of vertebrate genome structure, function and evolution. The chicken shares most of its biology with mammals but, being evolutionarily distinct, it serves as a useful resource for the discovery of a wide range of biological processes [1]. The recent release of a draft chicken genome sequence has substantially increased our understanding of its genomic landscape and provided additional insight into the comparative analyses of the chicken and mammalian genomes [2]. The chicken has a relatively small genome of approximately 1200 Mb, roughly threefold smaller than that of mammalian genomes. Surprisingly, less than 9% of the chicken genome contains transposable elements, compared with 40 to 50% in most mammals [3]. It is not yet known whether the low density of repetitive DNA elements in the chicken is due to low transposable element activity or to a lack of active retrotransposons in the chicken genome. The mechanisms that control the activity and copy numbers of chicken transposable elements remain unclear.

The most abundant transposable elements in the chicken genome (approximately 80%) belong to the CR1 families of LINE retrotransposons (Chicken Repetitive 1 Elements or CR1). The CR1 element resembles the mammalian L1 element in having a 5'UTR, followed by two open reading frames (ORFs) and a 3'UTR. A full-length CR1 element is approximately 4.5 kb in length. ORF1 has potential to encode a nucleic acid-binding protein of approximately 36 kDa containing a zinc-finger like motif; however, the exact function of ORF1 is not yet known [4]. ORF2 encodes a protein of approximately 95 kDa with critical endonuclease and reverse transcriptase activities as found in mammalian L1s, and is responsible for replication of the element. The majority of CR1 elements are truncated and mutated at their 5'ends, which is required for their transcription; nonetheless, there are a few copies of CR1 elements potentially capable of retrotransposition in the chicken genome [5,6]. Remarkably, the 3'UTR sequences of all CR1 subfamilies lack polyadenylated tails and instead contain two to four copies of a unique 8 bp (ATTCTRTG) repeat [4,6]. Interestingly, polyadenylated tails in mammals are potential substrates for L1 reverse transcriptase, probably explaining the abundance of processed pseudogenes in mammals compared with the chicken genome.

Little is known about the activity and function of chicken CR1 elements. The first draft of chicken genome reveals that at least 11 CR1 elements have the potential to produce functionally active elements, implying a role for these elements in shaping the chicken genome [3,4]. To date, more than 21 CR1 elements have been deposited in RepBase (2005). The chicken genome probably contains around 26,650 copies of the CR1 element (on the basis of BLASTN searches against the CR1 consensus sequence (accession no. U88211)) scattered throughout the genome. Despite this high number, it has been a puzzle as how these elements are transcribed and maintained in the chicken genome [3,4]. Given the low density of interspersed repeats in the chicken, mechanisms must exist to control the activity of CR1 retrotransposition. It remains to be elucidated how the copy number of transposons is controlled in the chicken genome and to what extent RNAi-mediated silencing mechanisms contribute to the activity of CR1 elements.

RNAi silencing machinery is highly conserved across eukaryotes. It may have initially evolved to defend the genome against invading transposable elements and other genomic parasites [7]. Recent studies in *Caenorhabditis elegans *[8], *Drosophila *[9,10] and cultured human cells [11,12] have shown that knockdown of RNAi machinery increases transcript abundance of retrotransposable elements. These findings all suggest that there is ongoing control of retrotransposon proliferation in the host [13]. However, it is not clear whether such controlling mechanisms of retrotransposons are currently active in the chicken genome. To explore this hypothesis, we knocked down Dicer gene expression in chicken DT40 cells and investigated the effects of siRNAs on CR1 transcripts. Here, we show that control of chicken CR1 is independent of the chicken RNAi machinery. The depletion of chicken Dicer did not result in increased expression of chicken CR1 elements. However, when we introduced human L1 elements into DT40 cells, in which Dicer expression was reduced, a cell culture-based retrotransposition assay showed a marked increase in L1 expression and retrotransposition activity but not of endogenous chicken CR1 transposons.

## Results

**Figure 1:**
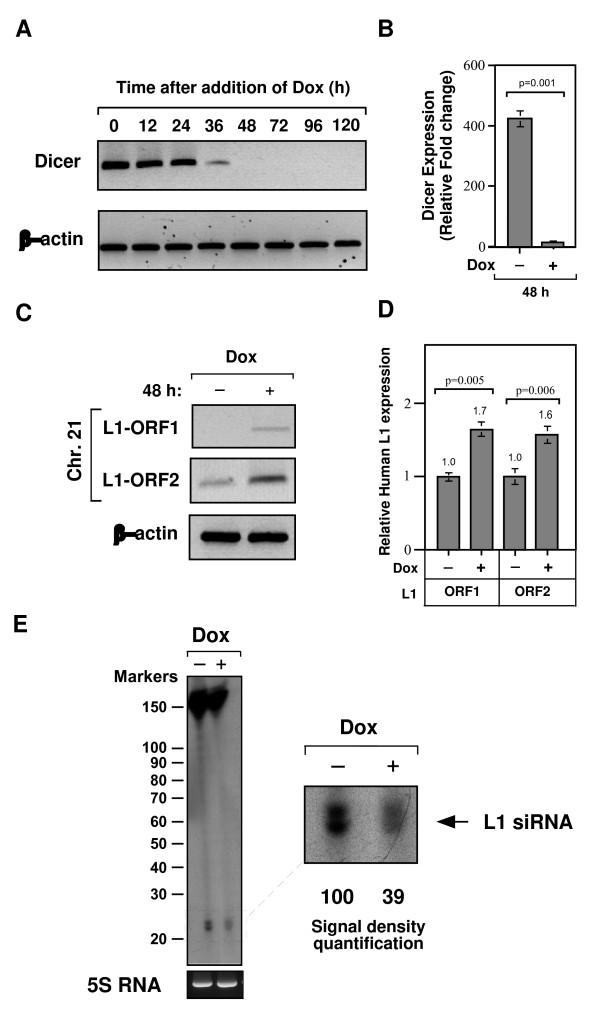
**Conditional loss of Dicer function in chicken DT40 cell line**. **(A) **Western blot analysis of Dicer-deficient whole-cell extracts with anti-Dicer antibody at the indicated times after addition of 2 μg/ml doxycycline (Dox). Equal amounts of extracts (approximately 20 μg) were resolved by SDS-PAGE and equal loading was confirmed by western blot analysis with an anti-β-actin antibody. **(B) **Quantitative real-time RT-PCR analysis of Dicer mRNA was determined in Dicer^+ ^cells (Dox-) and Dicer-deficient cells (Dox+). Plotted are values (in arbitrary units) for abundance of Dicer transcripts normalized to levels of chicken β-actin transcripts, a housekeeping gene. **(C) **The L1 transcripts derived from L1 elements of human chromosome 21 were detected in Dicer-deficient cells by strand-specific RT-PCR with primers specific for the L1's ORF1 and ORF2 sequences. Chicken β-actin served as an internal control. **(D) **Quantitative real-time RT-PCR analysis of human L1 mRNA was determined in the presence (Dicer-deficient) or absence of Dox (control cells). Relative fold change of L1's ORF1 and ORF2 mRNA levels after depletion of Dicer for 48 h was determined by normalizing the data with chicken β-actin gene. Error bars show s.d (*n *= 4). **(E) **Northern blot analysis of the human L1-specific siRNAs in chicken DT40 cells. The small RNAs extracted from control DT40 (Dox-) or Dicer-depleted DT40 cells (Dox+) were probed with L1 sequence and its signal density was quantified (see in insert). Signal density of control value was considered as 100%.

A key step in the RNA silencing pathway is the cleavage of double-stranded RNA precursors by Dicer to produce functional siRNAs. In a number of organisms, siRNAs have been implicated in silencing retrotransposable elements [7,14]. To understand the biological function of the RNAi pathway against the chicken CR1 elements, we used a conditional loss-of-Dicer function in a chicken-human hybrid DT40 cell line that containing a single copy of human chromosome 21 [15]. A conditional knockout of Dicer was generated in which a tetracycline-repressible promoter controlled the expression of Dicer. In the presence of doxycycline (Dox), a gradual loss of Dicer protein was observed, with complete loss 48 h after addition of Dox (Figure 1A). Quantitative RT-PCR with primers specific for chicken Dicer revealed that mRNA levels were up to 92% lower in Dox-induced DT40 cells than in the control cells (Figure 1B; *P *= 0.001). Moreover, the Dicer-deficient chicken cells survived for up to 6 days and their phenotype was almost indistinguishable from that of wild-type DT40 cells (data not shown). To further confirm the loss of Dicer function, we analysed the aberrant accumulation of transcripts from α-satellite DNA repeats in Dicer-deficient DT40 cells (see Additional file 1, Figure S1). Consistent with previous research [15], loss of Dicer in chicken cells resulted in increased levels of transcripts from α-satellite sequences through the disruption of Dicer-related RNAi silencing machinery.

### Upregulation of mammalian L1 transcripts in Dicer-deficient chicken cells

Dicer-mediated gene silencing is an evolutionally conserved system across eukaryotes. Previous studies in cultured human cells have identified that RNAi machinery elicited by the antisense transcripts of L1 retrotransposons can suppress L1 expression and retrotransposition efficiency [11]. To demonstrate the direct role of chicken Dicer in transposon silencing, and also to rule out the possibility that chicken may have multiple Dicer enzymes (as seen in *Neurospora *and *Aspergillus *[16,17]) that are redundantly responsible for transposon-specific silencing, we used human chromosome 21 as a source of mammalian L1 elements in chicken-human hybrid DT40 cells. This system has two unique advantages: first, it allows us to study L1 expression from a natural chromosomal environment rather than from transfected plasmids; second, as human L1 sequences are completely different from chicken CR1 elements, it is easy to identify the transcript abundance of L1 retrotransposons by quantitative RT-PCR analysis without interference from chicken CR1 transposable elements.

Human chromosome 21 contains 65 copies of a full-length L1 element determined by TBLAST and L1Base searches [18] using the consensus sequence of the active L1 element (accession no. AF148856). Although the majority of predicted L1 elements contain both ORF1 and ORF2, all but one of them carry mutations and frame-shift interruptions or in-frame stop codons. Only one copy of the L1 element in human chromosome 21 (position 17669229 – 17676620) appeared to be functional with an ability to produce both ORFs active proteins. Using real-time quantitative RT-PCR, we first compared the abundance of L1 transcripts in Dicer-deficient and control DT40 cells. Remarkably, Dicer knockdown in chicken cells resulted in a significant increase (around 1.6-fold) in the levels of both ORF1 and ORF2 transcripts derived from L1 element of human chromosome 21 (Figure 1D; *P *= 0.005 and *P *= 0.006 for ORF1 and ORF2, respectively). In addition, we also confirmed the increased expression of L1 transcripts upon loss of Dicer by strand-specific RT-PCR analysis (Figure 1C), suggesting that Dicer may be required for silencing of human L1 retrotransposons. These data also imply that the Dicer-mediated RNAi silencing pathway is functional in chicken DT40 cells and enables them to control the levels of human L1 retrotransposons.

Since Dicer is a crucial component in the RNAi pathway, which processes dsRNA precursors to functional siRNAs, loss of Dicer may contribute to an increased expression of L1 transcripts due to the absence of, or poorly processed, siRNAs against L1 elements. An equally important possibility is that loss of Dicer also affects miRNA processing, and thus the increased expression of L1 transcripts in the absence of Dicer could be an effect of miRNA dysregulation that targets L1 expression directly, or indirectly through miRNA-mediated regulation of cellular factors that suppress L1 expression. Although bona fide siRNAs or miRNAs that control the human L1 elements are yet to be isolated, a previous study in cultured human cells [11] has shown that the Dicer knockdown or deletion of L1's antisense promoter increases the levels of L1 expression, suggesting that the siRNAs derived from the active L1 promoter control L1 expression. To confirm that the Dicer knockdown in chicken cells affects L1-derived small RNAs processing and thus the increased expression of L1 transcripts, we analysed the accumulation of small RNAs in control and Dicer-deficient DT40 cells (Figure 1E). Northern blot analysis shows reduced accumulation of L1-specific siRNAs in Dicer-depleted cells (up to 61 ± 3% as measured by signal density quantification) compared with control cells, but not their complete absence, even though the level of Dicer was almost completely lost 48 h after addition of Dox. It is not clear whether the leftover small RNAs in Dicer-depleted cells are pre-existing siRNAs or poorly processed siRNAs upon the loss of Dicer. Nonetheless, this study shows that the reduced levels of L1-specific small RNAs in Dicer-deficient cells correlate with upregulation of the human L1 transcripts.

### Dicer knockdown activated mammalian L1 retrotransposition

**Figure 2:**
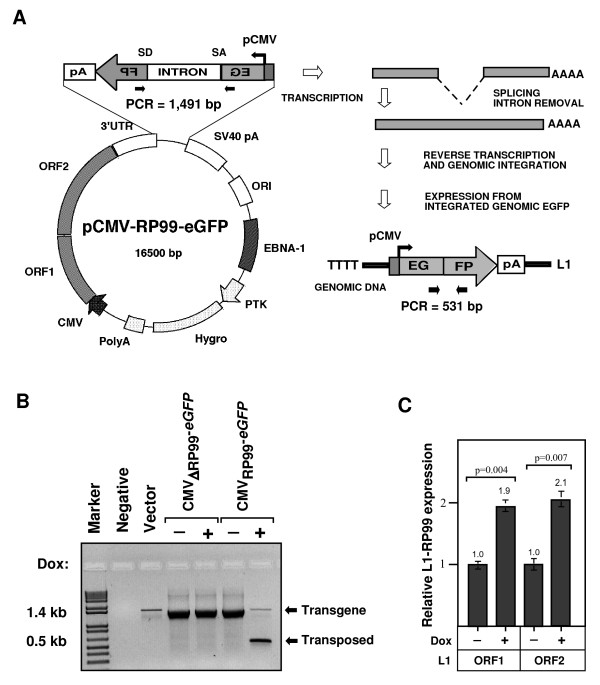
**Chicken DT40 cell culture-based assay for L1 retrotransposition**. **(A) **Schematic of the human L1-eGFP vector. The L1 transcription is driven by a CMV promoter in addition to the L1 5'UTR. The human L1 retrotransposon contains an intron-interrupted EGFP reporter in the 3'UTR region with its own promoter and polyadenylation signal. The EGFP cassette is in antisense orientation relative to L1. Only when EGFP is transcribed from the L1 promoter, spliced, reverse-transcribed and integrated into the genome does a cell become GFP-positive. As a negative control, inactive L1 (pCMV-ΔRP99-eGFP) that contained two missense mutations in ORF1 that abolish retrotransposition was used. Arrows depict the location of the geno-5 (left) and geno-3 (right) primers used in the PCR assay shown in below. SD = splice donor; SA = splice acceptor. **(B) **Detection of L1 retrotransposition events in chicken cells. The geno-5 and geno-3 primers that flank the intron in GFP were used for PCR and analysed on a 1.2% agarose gel. PCR products of 1.49 kb (corresponding to the intron-containing transgene) and approximately 0.5 kb that lacks the 909 bp intron (corresponding to the transposed insertion) are shown. Negative, genomic DNA from wild-type DT40 cells; Vector, 5 ng plasmid DNA; Marker, 1 kb-plus DNA marker (Invitogen). **(C) **Quantitative RT-PCR analysis of the L1 transcript of human L1 elements in control (Dox-) and Dicer-deficient (Dox+) cells after addition of 2 μg/ml Dox for 72 h. Data are normalized to that of chicken β-actin transcripts. Error bars show s.d. (*n *= 6).

To further confirm that the Dicer is indeed required for controlling human L1 expression and retrotransposition, we introduced L1 expression cassettes harbouring a retrotransposition indicator for cell culture-based assay [11,19]. This cassette consists of a full-length human L1 tagged at its 3'UTR with an antisense enhanced green fluorescent protein (EGFP) gene, which is driven by a cytomegalovirus (CMV) promoter (Figure 2A). The EGFP gene is disrupted by a γ-globin intron in the same orientation as the L1 transcript. This arrangement ensures that EGFP expression occurs only after L1 transcription, splicing of the intron, reverse-transcription, and insertion of the L1 copy back into chromosomal DNA (that is, after the retrotransposition event).

The chicken DT40 cells were electroporated with an active L1 element (pCMV-RP99-eGFP) and assayed for EGFP expression in the presence or absence of Dox. An inactive L1 (pCMV-ΔRP99-eGFP) that contained two missense mutations in ORF1 [19] to abrogate retrotransposition activity was used as a negative control. Cells expressing L1 were screened for EGFP expression by flow cytometry in order to compare the L1 retrotransposition events between Dicer-deficient and control DT40 cells. EGFP-positive cells were not detected in any of the control cells, even after several passages; PCR analysis also confirmed the absence of the retrotransposed or spliced EGFP in control cells (Figure 2B). This observation suggests that no retrotransposition events occurred in the chicken cells that contained intact Dicer and RNAi pathways. Strikingly, Dicer knockdown in chicken cells resulted in a significant increase in EGFP-positive cells (0.8 ± 0.07%) after 48 h addition of Dox, indicating that Dicer is indeed required for controlling the expression of human L1 elements. PCR analysis of chicken genomic DNA also confirmed the splicing of the intron in the EGFP gene in Dicer-deficient cells; these cells remained EGFP-positive for 5 days in culture and then ceased to proliferate upon loss of Dicer and subsequently died before 7 days by apparent cell death or apoptosis [15]. Furthermore, we also observed a correlation between the relative abundance of L1 transcripts and retrotransposition events by real-time quantitative RT-PCR analysis (Figure 2C). The level of L1 transcripts was almost doubled after Dicer knockdown (*P *= 0.004 and *P *= 0.007 respectively for L1's ORF1 and ORF2) suggesting that chicken Dicer gives rise to an inhibitory effect on retrotransposition by reducing the levels of L1 transcripts, most probably via L1-derived siRNAs.

### Dicer is not responsible for silencing of chicken CR1 transposons

**Figure 3:**
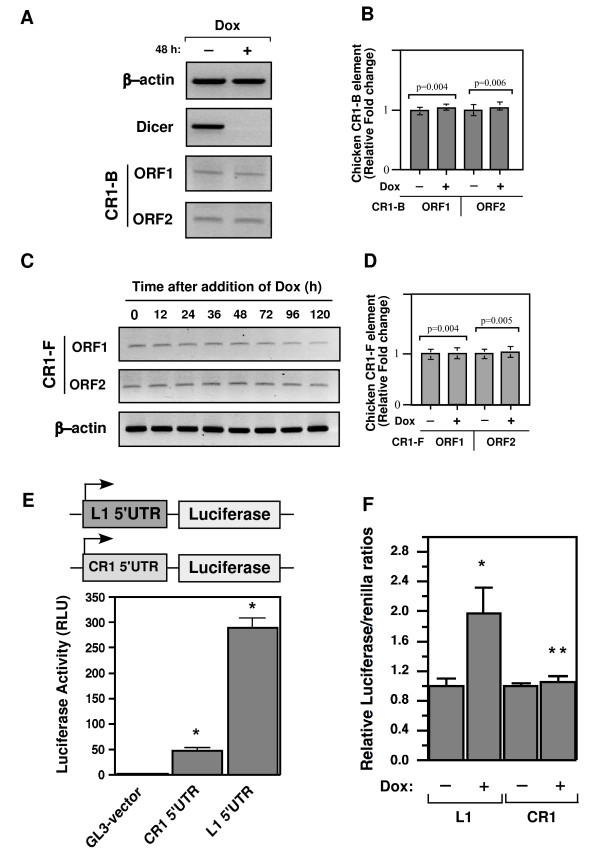
**Dicer knockdowns do not activate endogenous CR1 transcripts in Dicer-deficient DT40 cells**. **(A, C) **Strand-specific RT-PCR targeting the sense message of CR1 elements was performed to determine the relative levels of CR1-B and CR1-F elements by primer sets that detect CR1's ORF1 and ORF2 from Dicer^+ ^(Dox-), and Dicer-deficient (Dox+) cells. Digital photographs (negative image) of agarose gel analyses of the RT-PCR products are shown including the transcripts of CR1-B, CR1-F, Dicer, and chicken β-actin (internal control). **(B, D) **Quantitative real-time RT-PCR analysis of CR1-B and CR1-F transcripts were determined after normalizing the data with chicken β-actin. Error bars show s.d. (*n *= 6).**(E) **A schematic of the constructs used for the luciferase reporter assay is shown in the top panel. Luciferase reporter activity of CR1 5'UTR compared with L1 5'UTR after normalized for Renilla luciferase reporter is shown in bottom panel. In this assay, the negative control GL3 vector was used. Error bars show s.d. *P < 0.001. **(F) **Relative luciferase activity of CR1 and L1's 5'UTR in chicken DT40 cells measured in the presence or absence of Dicer. All activities were normalized to that of Renilla luciferase reporter and arbitrary units of each relative luciferase activity converted into the percentage. *P < 0.005 and **P < 0.001.

If siRNAs were responsible for controlling the copy numbers of chicken CR1 transposons, one would expect that depletion of siRNAs by Dicer knockdown would lead to a higher level of endogenous chicken CR1 transcripts in the chicken genome. To examine whether chicken Dicer is responsible for processing siRNAs acting against the chicken CR1 transposons, we used both strand-specific RT-PCR (targeting the sense message) and real-time quantitative RT-PCR to assess the transcript levels of CR1-B elements in DT40 cells in the presence or absence of Dox for 2 days. Unexpectedly, there was no significant difference in transcript accumulation of chicken CR1 elements in Dicer-deficient cells compared with control DT40 cells (Figure 3A). The mRNA levels of both ORF1 and ORF2 of CR1-B elements in Dicer-deficient and control DT40 cells were close to background levels, as determined by real-time quantitative RT-PCR (Figure 3B; *P *= 0.004 and *P *= 0.006 for ORF1 and ORF2, respectively). Consistently, regardless of whether Dicer was weakly or strongly depleted, Dicer knockdown did not increase CR1 mRNA levels (data not shown). To further confirm this finding, we analysed transcript levels of the functionally active CR1-F element in chicken chromosome 6 (positions 2162180 – 2166909) [4]. Again, we found a basal level of CR1 transcription that was similar in both control and Dicer-deficient DT40 cells (Figure 3D). These data were further confirmed by strand-specific RT-PCR analysis targeting the sense message of CR1 elements (Figure 3C), suggesting that the Dicer-related RNAi machinery may not be responsible for controlling the endogenous chicken CR1 elements.

Next, to determine whether the CR1 elements are able to produce any transcription, we transfected the chicken DT40 cells with luciferase reporter constructs whose expression is driven by either functionally active CR1-B 5'UTR or L1 5'UTR as a promoter and performed the Dual-Luciferase assay with the Renilla luciferase plasmid. In this way, we measured promoter activity in the presence or absence of Dicer. As expected, transcript level of the human L1 5'UTR promoter in Dicer-depleted DT40 cells was around twofold greater than that of control cells but not chicken CR1-B 5'UTR promoter, whose level of expression was almost comparable to that of the control DT40 cells (Figure 3F). This observation suggests that the chicken CR-1 elements may not be controlled by the Dicer-mediated RNAi pathway. Interestingly, when we reprobed the northern blot of small RNAs against the mixture of synthetic sense-strand CR1-B 5'UTR sequences, we were not able to detect any CR1-specific antisense transcripts, regardless of whether the Dicer was present or absent (data not shown). This suggests that the CR1 promoter may not produce any siRNAs like the human L1 5'UTR promoter. Remarkably, according to the luciferase reporter assay, L1 promoter seems to be at least sixfold stronger than that of the CR1 element (Figure 3E), indicating the existence of major differences between chicken CR1 and other mammalian L1 elements in their 5'UTRs, which conceivably function as promoters.

### Very few CR1 5'UTR promoters are functional

Evidence from RNAi silencing of human L1 retrotransposons suggests that the first step for RNAi machinery is synthesis of dsRNAs from the transcriptionally active 5'UTRs of retrotransposons [11,14,20]. The human L1 5'UTR is known to contain a bidirectional promoter (both sense and antisense promoters) that leads to the production of dsRNAs, which are processed by Dicer into siRNAs. To investigate the possibility that chicken 5'UTRs might transcribe dsRNAs, perhaps through DNA hypomethylation, we analysed all putative 5'UTRs of chicken CR1 elements identified through literature and database searches. A BLASTN search against a CR1 consensus sequence (GenBank Acc. U88211) suggests that the chicken genome probably contains around 26,650 copies of the CR1 element scattered throughout the genome. To identify potentially active CR1 elements with intact promoters, the 5'UTRs of the previously identified active F and B subfamilies [4,6] were initially used for a Blast search against the chicken genome. About 148 UTRs (136 from the CR1-F and 12 from the CR1-B subfamilies) were identified and used in the promoter analysis. Surprisingly, except for 15 UTRs (seven CR1-F and eight CR1-B), the vast majority of CR1 5'UTRs (>90%) are found as short fragments (less than half of the expected size) and are severely truncated or mutated to varying degrees. Notably, most of the mutations or deletions occurred at putative transcription factor binding sites as determined by TRANSFAC searches [21] against the promoter sequences of the CR1 elements. Unlike the functionally active CR1 sequences, these elements do not contain *cis*-acting elements or putative promoter-like sequences upstream of the start codon, suggesting that the overwhelming majority of chicken CR1 elements do not have the necessary functional promoter sequences for initiation of RNA transcription.

**Figure 4:**
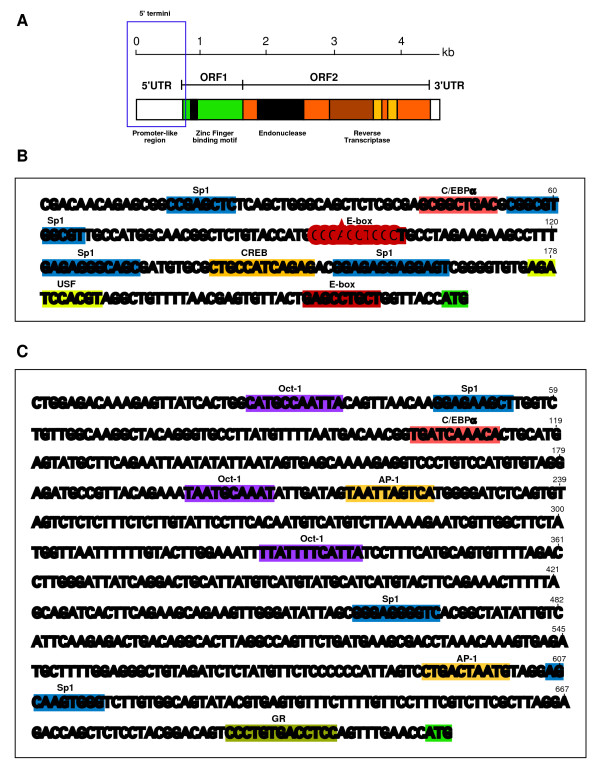
**The chicken CR1 subfamilies have a distinct 5'UTR sequences**. **(A) **Diagram of the approximately 4.5 kb full-length chicken CR1 element structure. The 5'UTR promoter region analysed is boxed. **(B) **The 5'UTR of CR1-B subfamily contains sequences similar to those found in the sequence from turtle, pufferfish and other reptiles CR1 elements. E boxes and other binding sites for transcription factors found in this region are shown in shaded regions. The putative transcriptional start codon is highlighted in green. **(C) **The 5'UTR sequences of the CR1-F subfamily. The shaded regions show the binding sites for several transcriptional factors including Oct-1 and RXR-β sites.

The 5'UTR of the potentially active CR1-B element is approximately 240 bp long and contains two putative E boxes, the *cis*-element for binding of the basic helix-loop-helix family of proteins. E boxes have been noted as an obvious feature of vertebrate CR1-elements [22]. In addition to the E boxes, other potential binding sites for C/EBP, USF and Sp1 are also found in the promoter sequence of CR1-B element (Figure 4B and Additional file 1, Table S4). Unlike other vertebrate CR1-elements such as those in the turtle and puffer fish [22,23], there is no obvious evidence of a 32-bp direct repeat sequence within the CR1-B promoter sequence. Several deletions or insertions, ranging from several nucleotides to several dozen nucleotides, were found in these regions of the CR1-B direct repeats suggesting that this region might have undergone frequent recombinational events. Direct repeat sequences are often thought to be involved in dsRNA production, which are processed by Dicer into functional siRNAs. Interestingly, the 5'UTR sequence of CR1-F element shows no resemblance to the 5'UTR of CR1-B elements. Unlike the CR1-B element, there is no sequence corresponding to canonical E boxes (Figure 4C). Promoter analysis of the CR1-F elements shows the presence of at least three regions corresponding to the binding of Oct-1/AP-1 (Fos/Jun activating protein-1) core elements within the region of promoter. In addition, a potential binding site for GR (glucocorticoid receptor) is also located at upstream of the start codon. GR is a zinc-finger DNA-binding protein likely to be involved in the activation of CR1-F elements through a Fos/Jun complex similar to that found in the chicken vitellogenin and ovalbumin genes [24]. This observation raises the possibility that cellular transcription factors that bind to these genes might act in concert to regulate the expression of CR1-F elements.

### Sequence divergence of 5'UTR within each CR1 subfamily

**Figure 5:**
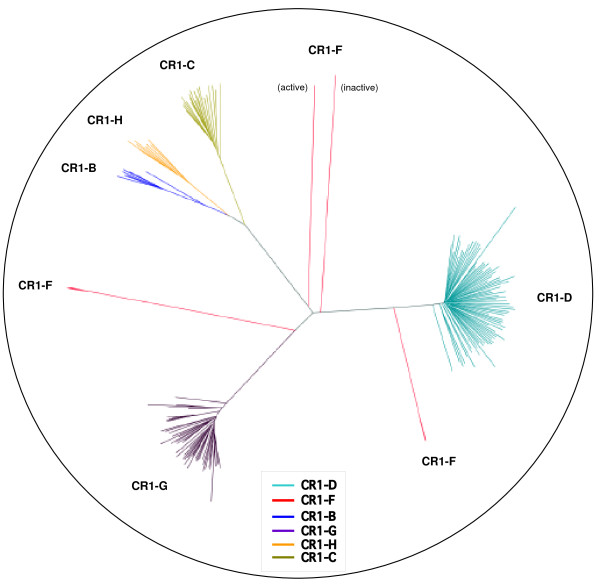
**Phylogenetic analysis of the 5'UTR promoters of all chicken CR1 subfamilies**. The tree is constructed based on sequence diversity of all 5'UTRs, including functional and non-functional CR1 elements, using the neighbour-joining method. Each CR1 subfamily is shown in different colours, and CR1-F subfamily with more than one branch is shown in the same (red) colour.

The intact and functionally active CR1 elements identified thus far belong to the CR1-B and CR1-F subfamilies. Our study shows that the 5'UTR promoter sequence of B and F elements are distinct and unrelated to each other, suggesting the existence of sequence diversity in the 5'UTR promoters in the chicken genome. The relationships among the 5'UTRs of all other CR1 subfamilies (including the previously described CR1 elements C, D, E, and H) are poorly understood. To evaluate the biological significance of all these CR1 promoters, we downloaded the 5'UTR sequences of all CR1 subfamilies in the chicken genome and aligned them using ClustalX (see Additional file 1, Tables S2 and S3). Sequences that contained large truncations or deletions in the alignment of the 5'UTR sequences were removed, resulting in a final set of 169 sequences that were used for the construction of a phylogenetic tree (Figure 5). The 5'UTRs of chicken CR1 elements cluster into six distinct subfamilies. The largest group of 5'UTRs belongs to subfamilies D and G (with 75 and 51 elements, respectively) scattered throughout the chicken genome. Except for a very few copies of B and F subfamily members, none of the CR1 elements representing the C, D, G, and H subfamilies contain sequences that might provide functionally active CR1 elements (data not shown). This indicates that they are not retrotransposition competent. The most notable finding that emerged from our sequence analysis is that the 5'UTR sequence of the functionally active CR1-F element has diverged from that of the non-functional CR1-F elements (Additional file 1, Table S5) indicating the existence of distinct promoter sequences for functional elements within the CR1 subfamily of the chicken genome.

### Functional CR1 elements are rare in the chicken genome

Next, we analysed whether the intact 5'UTR promoter contains the genomic DNA sequence of full-length ORF1 and ORF2 sequences. For this analysis, the consensus protein sequence of ORF1 was initially used in a protein BLAT search against the chicken genome. The regions that showed similarity were isolated along with 6 kb of their flanking sequences. In a second step, the resulting genomic DNA sequences were screened for the presence of an intact 5'UTR promoter. We identified only six full-length CR1 elements with intact promoters (four belonging to the CR1-F subfamily and two to the CR1-B subfamily) that have a size of >4,200 bp; the others were severely truncated at either the 5' or 3'region of ORFs. Out of four CR1-F elements, three were found to contain intact promoters capable of RNA transcription, but these elements are defective in the coding region of ORF1 and ORF2 due to frame-shift mutations (see Additional file 1, Table S6). Only one copy of the CR1-F element found on chromosome 6 with an intact promoter (chr. 6: position 2161469 – 2166909) appears to be functionally active with the ability to produce both ORF proteins. Its sequences are also in agreement with previous report of a functional 'mother' CR1 element [4]. Similarly, out of two intact CR1-B elements, only one copy a of CR1-B element with intact promoter and ORFs for both proteins was found on chromosome 5 (position 5715277 – 5719735). This element is 98% identical with the consensus sequence of CR1-B (accession no. U88211), whereas the other CR1-B element on chromosome 2 (51121971 – 51126431) was truncated at ORF1. Interestingly, two additional CR1-B elements on chromosomes 5 and 2 contained intact ORF regions (Additional file 1, Table S6) but these elements do not contain promoter sequences that can serve as an initiation point for transcription. In summary, the overwhelming majority of chicken CR1 elements appear to be non-functional and thus unable to replicate. Nonetheless, our findings suggest that there is at least one copy of the CR1-F and B subfamily still potentially capable of active in the chicken genome.

## Discussion

Silencing the activity of retrotransposable elements is important for maintaining genomic integrity and stability [25]. It is commonly thought that siRNAs derived from repetitive elements can protect the genome against transposable elements. Supporting this notion, the first evidence in mammals [11] shows that siRNA derived from both strands of active L1 sequences control expression of L1 retrotransposons by Dicer-mediated RNAi silencing pathways. It has also been shown that knocking down Dicer expression in human cells can lead to a marked increase in retrotransposition activity as well as accumulation of L1 transcripts, providing a direct link between the RNAi machinery and suppression of transposable elements. A single copy of the Dicer gene is also present in chicken and other vertebrates [26,27], although the biological functions of Dicer-mediated silencing pathways in the chicken are not yet fully understood. Notably, until now there has been no direct evidence of an involvement of the chicken Dicer enzyme in controlling the activity of endogenous chicken CR1 transposons.

In our current analysis, we evaluated the contribution of the Dicer-mediated RNAi machinery to control of chicken CR1 elements. In contrast to mammals, Dicer knockdown in chicken cells did not result in increased expression of endogenous CR1 elements. However, when we introduced transcriptionally active human L1 elements into chicken cells in which Dicer expression was depleted, a significant increase in the accumulation of L1 transcripts, as well as retrotransposition, was observed. The ability of chicken Dicer to perform RNAi silencing of mammalian transposons but not chicken CR1 elements highlights a major difference between the chicken CR1 and mammalian transposable elements. The response of chicken RNAi machinery against exogenous L1s seems to be more subtle than that of some other eukaryotes such as mouse, *C. elegans *and *Drosophila*, whose genomes can rapidly accumulate retrotransposon transcripts and acquire a mutator phenotype through disruption of RNAi pathway [20,28]. This 'lying in wait' response of the chicken against human L1 elements is reminiscent of the RNAi response against the LINE-like Tad transposon in *Neurospora *[29,30]. Interestingly, like the chicken CR1 elements, most of the Tad transposons are inactive in *Neurospora *due to mutation and truncation. However, upon introduction of transcriptionally active Tad elements, siRNAs against Tad elements are produced, prompting the activation of an RNAi-mediated silencing pathway. Thus, perhaps one role of the RNAi machinery is to permit a rapid and potent response to the sudden activation of L1 retrotransposons.

There are several possible explanations for our failure to detect expression of endogenous CR1 transposons in Dicer-deficient chicken cells. In one plausible model, we considered the possibility that additional chicken Dicer enzymes might be redundantly employed in CR1 transposon control pathways, similar to that seen in *Neurospora *in which two Dicer enzymes are mutually able to control Tad transposons [17]. Recent evidence from *Drosophila *and *Arabidopsis *has also demonstrated that different Dicer homologues can provide distinct substrate specificity [31]. However, this does not seem to be the case in chicken cells, which are known to contain a single copy of Dicer on chromosome 5 [27]. In addition, Dicer deficiency in chicken DT40 cells has already been shown to cause defects in heterochromatin formation and aberrant accumulation of transcripts from α-satellite sequences through disruption of RNAi-mediated silencing pathways [15]. Moreover, our current data suggest that Dicer-deficient chicken cells retain their ability to control mammalian L1 retrotransposon expression.

Alternatively, as we see in mammals, activation of the RNAi machinery may depend on initial transcription of retrotransposons to produce dsRNAs, which serve as substrates for Dicer processing into siRNAs [11]. Surprisingly, most of the chicken 5'UTRs (>90%) are found to be severely truncated and mutated in the promoter regions of CR1 elements; nonetheless, there remain a very few copies of CR1-F and CR1-B elements still potentially capable of retrotransposition. Our data show that except for one copy of the CR1-F and CR1-B elements, almost none of the chicken CR1s contain putative promoter-like sequences upstream of the transcriptional start codon, indicating that almost all chicken CR1 elements are not functionally active. Only one functional CR1-F element with an intact promoter sequence and ORFs for both proteins is found, on chromosome 6 (Repbase 2005) and is flanked by 5-bp target site duplication. Similarly, only one copy of the CR1-B element identical with the consensus sequence of CR1-B (accession no. U88211) is found on chromosome 5. This suggests that functionally active CR1 elements are very rare in the chicken genome. Of course, it is possible that most of the CR1 elements have lost their activity during the course of evolution and become non-functional. The drastic reduction of intact CR1 copies and the lack of many active CR1 elements in the chicken genome suggest that chicken CR1 elements may possibly be approaching extinction.

A recent study in mammalian oocytes suggests that around 1,000 molecules of dsRNA are required to trigger Dicer-mediated RNAi silencing pathways [32,33]. Production of such high levels of dsRNA is likely to correlate with the level of RNA expression. Absence of large numbers of functionally active CR1 promoters is probably one of the reasons that even if a few copies of CR1-F and CR1-B do function, they are not sufficient to trigger Dicer-mediated silencing. Consistent with this, the present study shows that the chicken CR1-B promoter is much weaker than that of the human L1 promoter, by factor of sixfold. Interestingly, the evolution of CR1 in the chicken genome seems to differ from that of L1 in mammals; unlike the single L1 lineage in mammals, our analysis shows that the chicken CR1 elements form six distinct subfamilies (CR1-B, C, D, F, and H) that are considerably diverged from each other in their 5'UTR regions. These results are also in agreement with a previous report of the 5'UTR sequences of chicken CR1-B and CR1-F elements [4,6]. Given the low degree of sequence homology in the 5'UTRs of CR1 elements, if any siRNAs are produced from a single CR1 locus, they would not have the potential to target every CR1 transcript for RNAi-mediated control. Indeed, our data suggest that control of the activity of chicken CR1 transposons is not mediated by Dicer-related pathways (probably because of a lack of transcriptionally active CR1 elements), but by other silencing pathways.

It seems likely that the chicken has multiple mechanisms to ensure suppression of CR1 silencing, similar to what is seen in mouse oocytes in which retrotransposon silencing is carried out by both siRNA and pi-interacting RNA (piRNA) pathways [34]. One plausible mechanism is Dicer-independent piRNAs. Unlike siRNAs, piRNAs do not depend upon Dicer [7,35] and do not seem to be derived from dsRNA precursors of active retrotransposons [36]. Instead, they seem to arise from single-stranded RNAs that are transcribed from piRNA clusters. The nuclease activity of the Piwi proteins (PIWI and Aubergine orthologues) is required for piRNA processing. Recent studies in mice and *Drosophila *showed that mutations in Piwi proteins resulted in a loss of retrotransposon control [35,37], suggesting a function for piRNAs in retrotransposon control. piRNAs are highly abundant in germ cells but some evidence suggests that they are also active in somatic tissues [38] and are involved in retrotransposon silencing through chromatin modifications [13]. Given that piRNA production is not dependent on either Dicer activity or transcriptional activation of retrotransposons to produce dsRNA precursors, it is plausible that silencing of CR1 elements in the chicken might primarily occur through Dicer-independent piRNA pathways. An equally important possibility is cellular factors that can suppress the expression of CR1 elements in the chicken genome. Further studies are required to provide the mechanistic support for CR1 silencing machinery.

## Conclusion

The recent release of a chicken genome sequence revealed that less than 9% of the chicken genome contains retrotransposable elements. It is not known whether the low density of repetitive DNA elements in the chicken genome is due to a lack of active elements or if chickens employ small RNAi-mediated silencing pathways that control the activity and copy numbers of transposable elements. We analysed the loss of Dicer function on chicken endogenous CR1 elements with impaired RNAi machinery and found that the control of chicken CR1 elements is independent of the Dicer-mediated RNAi silencing pathway. In addition, our results strongly suggest that except for single copies of the CR1-F and CR1-B elements, almost none of the chicken CR1 elements contain the necessary promoter sequences for initiation of RNA transcription. Phylogenetic analysis shows the existence of sequence diversity at the 5'UTR promoters within each CR1 subfamily, indicating that they are not potential targets for controlling the activity of every CR1 transcript by siRNA pathway. Our results provide the first evidence that the low level of repetitive elements in the chicken genome is probably due to the absence of a large number of active CR1 elements.

## Methods

### Gene targeting and cell culture

The chicken DT40 cell line containing a chicken Dicer transgene under the control of a tet-repressible promoter (Dicer-deficient/tet^+^; 2–49 cells) was a kind gift from Tatsuo Fukagawa (PRESTO of JST, Mishima, Japan). Detailed descriptions of Dicer gene targeting and expression of constructs are given in [15]. The Dicer-deficient DT40 cells were maintained in PRMI 1640 (Gibco) with 2 mM L-glutamine, 10% fetal calf serum, 1% chicken serum, 50 μM β-mercaptoethanol, 2 mg/ml geneticin, 0.5 μg/ml puromycin (Sigma), and 1 mg/ml Zeocin (Invitogen). Expression of the tet-responsive Dicer transgene was suppressed by the addition of 2 μg/ml Dox (Sigma) to the culture medium.

### Plasmids and transfection

The human L1 expression vector, pCMV-RP99-eGFP and retrotransposition-defective vector, pCMV-ΔRP99-eGFP were created by inserting a CMV promoter in frame between *Not*I and *Sal*I restriction sites of pRP99-EGFP and pJM111-EGFP (obtained from HH Kazazian, University of Pennsylvania). For transfection, exponentially growing chicken DT40 cells (approximately 5 × 10^6^) were harvested and electroporated with 15 μg of pCMV-99RP-eGFP or pCMV-ΔRP99-eGFP at 250 V and 960 μF in a Gene Pulser II (Bio-Rad) as described by Honda *et al*. (2007) [39]. To examine the transfection efficiency in chicken DT40 cells, pDsRed-N1 (Clontech) was used as a reporter control. At 24 h after transfection, cells were equally split and cultured in the presence or absence of 2 μg/ml Dox at the indicated times. GFP-positive cells were analysed by sorting with a FACSVantage DiVa cell sorter (Becton-Dickinson). Each experimental group was transfected in triplicate and repeated twice.

### Retrotransposition assay

Genomic DNA was isolated from DT40 cells at 72 h after addition of Dox or from control cells using QIAamp DNA kit (Qiagen). PCR was carried out to examine the structure of the EGFP reporter cassette integrated in this genomic DNA with the Geno-5' and Geno-3' primers (see Additional file 1, Table S1). Amplifications were performed in 50 μl containing 1.25 U Taq polymerase, 2.5 mM MgCl_2_, 1× PCR buffer (Fermentas), 0.2 mM each dNTP, 5 μM of each primer and 500 ng of genomic DNA or 5 ng of plasmid DNA template. After an initial step at 95°C for 5 min, 35 cycles of amplification were performed (95°C for 30 s, 58°C for 15 s and 72°C for 2 min), followed by a final step at 72°C for 8 min. The amplified products were visualized on a 1.2% agarose gel. The genomic DNA of wild-type chicken DT40 cells was used as a negative control.

### Quantitative RT-PCR

Total RNA was isolated from the Dicer-deficient DT40 cells using an RNeasy Kit (Qiagen) and digested with TurboDNase-I (Ambion). A total of 2 μg of purified RNA was used in cDNA synthesis with 0.1 μg of random decamer primers using the RETROScript RT Kit (Ambion). The resulting cDNAs (fivefold serial dilutions of cDNA; 100, 20, 5, 1 and 0.2 ng per reaction) were used as templates for QPCR with the gene-specific primers (Additional file 1, Table S1). In this assay, performing cDNA synthesis in the absence of reverse transcriptase served as negative controls. QPCR was performed by using SYBR Green PCR master mix and 7900HT Thermal Cycler (Applied Biosystems) at typical amplification parameters (50°C for 2 min and 95°C for 10 min, followed by 40 cycles of 95°C for 15 s and 60°C for 1 min). The fold differences were determined by comparing the ΔC_T _value of each gene normalized with the chicken β-actin as a reference control for each reaction. Data generated was the average of three independent experiments with each experiment performed in triplicate, and analysed using the Relative Expression Software Tool (REST, version 2) [40].

### Strand-specific RT-PCR analysis

One microgram of purified total RNA was reverse-transcribed with 2 μM of antisense-strand (internal) primers (for targeting the sense message of transposons) and SuperScriptase II (Invitrogen) following the manufacturer's instructions. Negative controls included carrying out cDNA synthesis in the absence of reverse transcriptase and primers. All the primer sequences used in this assay are given in Additional file 1, Table S1. One microlitre of the resulting cDNAs was used as template for subsequent PCR analysis with the gene-specific primer sets. In these assays, the chicken β-actin primer was used as a reference control. The amplified products were visualized on a 2% agarose gel. The intensity of the DNA bands was also assessed with Gene-Snap software (Syngene, USA) following the manufacturer's instructions.

### Small RNA isolation and northern blot

Total RNA was extracted using Trizol reagent (Invitrogen) from control or Dicer-depleted chicken DT40 cells. About 100 μg of total RNA was subjected to enrichment of small RNAs by adding 50% PEG-8000 and 5 M NaCl to a final concentration of 5% and 0.5 M, respectively. The resulting supernatant (that is, the small RNAs fraction) was precipitated by ethanol along with 2 μg glycogen (Promega) and dissolved in 20 μl water. An equal amount of small RNA sample was electrophoresed in a 15% urea-PAGE gel and then transferred onto Hybond-N+ membrane (Amersham Pharmacia). L1 RNA probes were prepared by *in vitro *transcription using the Megascript T7 *in vitro *transcription kit (Ambion) according to the manufacturer's instructions. The resulting labelled dsRNA was hydrolysed to an average size of 50 nt by sodium bicarbonate treatments as described by Nolan *et al*. [29]. RNA size markers were prepared from Decade Markers (Ambion) following the supplier's instructions and approximately one picomole of radiolabelled size markers was loaded on a gel. For chicken CR1 elements, a mixture of three sense-strand oligonucleotides, each 70 bp in size covering most of the CR1-B 5'UTR region (see Additional file 1, Table S1), was used as probe after end labelling with T4 polynucleotide kinase (Fermentas). Hybridization and washing of the northern blot were performed as described elsewhere [29]. Signals were detected by exposure to Phosphor-Imager FLA-3000 system (Fuji, Tokyo) and density of the signal was quantified by Fuji film's multi Gauge software.

### Luciferase assay

The 5'UTR of human L1 elements was amplified by PCR from pRP99-EGFP and cloned into the reporter plasmid pGL3 (Promega) upstream of the luciferase gene. A synthetic double strand DNA sequence covering the entire region of 241 bp of the CR1-B 5'-UTR (chr. 5: position 5715277 – 5715518) was synthesized (Gene Work, Australia) and cloned into pGL3 at the *Sma*I site and confirmed by DNA sequencing. A Renilla luciferase vector, pRL-CMV (Promega) was used to correct the transfection efficiency. The chicken DT40 cells were cotransfected with the modified pGL3 firefly luciferase (under the control of either L1 5'UTR or CR1-B 5'UTR as a promoter) and the Renilla luciferase reporter plasmid. Firefly and Renilla luciferase assays were measured after 48 h in the presence or absence of 2 μg/ml Dox with the Dual-Luciferase Reporter assay system (Promega). Firefly activity was normalized to Renilla activity to control the transfection efficiency.

### Western blot analysis

Total protein was extracted from the Dicer-deficient 2–49 cells using the M-PER reagent (Pierce). About 20 μg of whole-cell extract was separated in a 10% SDS-PAGE gel, transferred onto a Protran Nitrocellulose membrane (Schleicher & Schuell), and probed with anti-Dicer antibody, DcR1 (1:500 dilution, Abcam) or anti-β-actin antibody, clone AC-15 (1:4000 dilution, Sigma). The membrane was then probed with secondary peroxidase-conjugated anti-mouse (1:5000 dilution, Silenus) for 1 h and visualized with an ECL detection kit (Amersham Biosciences).

### Computational analysis

For identification of the CR1 promoter sequences, the previously described repeat sequences of CR1 subfamilies [4,6,41] were used as query sequences in DNA BLAT or protein BLAT searches against sequences from the UCSC chicken genome [42] and Gallus GBrowse [43]. The sequences that aligned with the initial seed sequence were grouped with it if they were at least 75% similar over 50% of their length. The consensus sequences for each repeat subfamily were constructed based on multiple sequence alignment and neighbour-joining trees using ClustalX. The binding sites for transcription factors on each 5'UTR CR1 subfamily were analysed using the MATCH programs in the TRANSFAC 7.0 [20,44].

## Abbreviations

BLAT: blast-like alignment tool; CR1: chicken repeat 1; CMV: cytomegalovirus; Dox: doxycycline; EGFP: enhanced green fluorescent protein; GR: glucocorticoid receptor; L1 or LINE-1: long interspersed nuclear elements; ORF: open reading frame; piRNA: pi-interacting RNA; RNAi: RNA interference.

## Authors' contributions

DR designed and performed the experiments and prepared the manuscript. PE performed cell culture and FACS analysis. SL and SY performed computational analysis. All authors read and approved the final manuscript.

## Supplementary Material

Additional file 1 Supplementary Figure and Tables. Click here for file
