# Supplementary material for: Control of chicken CR1 retrotransposons is independent of Dicer-mediated RNA interference pathway
Source: BMC Biol. 2009 Aug 19;7:53. doi: 10.1186/1741-7007-7-53 (PMC2734521; doi:10.1186/1741-7007-7-53)
Supplement: Additional file 1 — Supplementary Figure and Tables. [file 1741-7007-7-53-S1.pdf]

Supplementary Figure Legends

Figure S1 shows aberrant accumulation of transcripts from α-satellite DNA repeats of human chromosome 21 correlates with Dicer-deficiency in chicken DT40 cells but not in control cells.

Supplementary Table Legends

Table 1 showing the primer sequences used in this study.

Table 2 provides the lists of 5'UTR of CR1 elements in the chicken genome.

Table 3 shows nucleotide alignments of CR1-1 subfamilies.

Table 4 contains the sequences and positions of predicated transcription binding sites on CR1 subfamilies (For details of potential binding sites for transcription factors in this figure legend, the reader is referred to the web link of this table).

Table 5 shows the 5'UTR promoter sequence of functionally active CR1-F element is diverged from non-functional CR1-F elements.

Table 6 provides the lists of full-length CR1-F and CR1-B elements and their positions in the chicken genome.

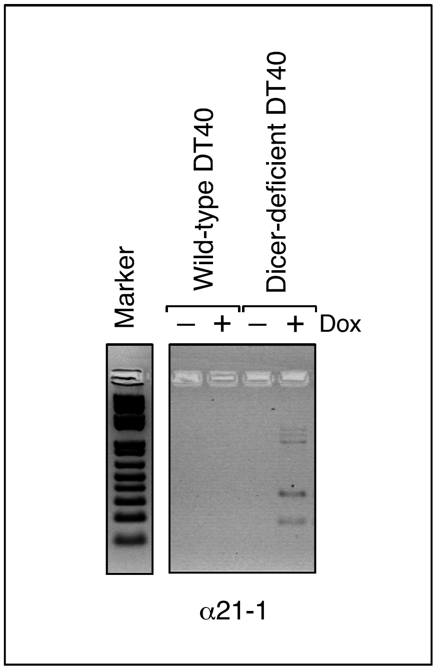

Figure S1: In the absence of dicer activity, Dicer-deficient chicken-human hybrid DT40 cells produces long RNA transcripts from centromeric repeats of human chromosome 21. RT-PCR analysis of α21-1 repeats was determined in wild-type DT40 and Dicer-deficient DT40 cells in the absence of (Dox-) or presence of Dox (Dox+) for 36 h. We used the human chromosome 21 satellite primers for this analysis as described by Ikeno et al. (1994) Hum.Mol.Genet. 3(8), 1245-1257.

Supplementary Table 1. Primer Sequences

Primer set	Purpose	Forward primer	Reverse primer
CHICKEN β-ACTIN	Gene-specific qRT-PCR of β-actin	CAGACATCAGGGTGTGATGGTTGG	GGGGTGTTGAAGGTCTCAAACATG
Actin-internal	Antisense of β-actin for strand-specific RT-PCR		GGCGTAGCCTTCATAGATGGGCA
CR1-B-ORF1	Gene-specific qRT-PCR of CR1-B (ORF1) family	AACTTGAGGGAGGGGTGAGTGAGA	CTCCATCAACTACCTGCCCCAGT
B-ORF1-internal	Antisense of CR1-B, ORF1 for strand-specific RT-PCR		GGTGGGTTTTTTGGATCCTCTCAGT
CR1-B-ORF2	Gene-specific qRT-PCR of CR1-B (ORF2) family	AAACTCCATGGGCAAAGATGGTG	GGCTCTCGACCTGTTCCTGACTG
B-ORF2-internal	Antisense of CR1-B, ORF2 for strand-specific RT-PCR		CGATGCCTCCCACATCTGGAGCAA
CR1-F-ORF1	Gene-specific qRT-PCR of CR1-F (ORF1) transposon	AGGTCAGCTCCACGCTTAAA	TTCTGTCAGCAACCTCATCG
F-ORF1-internal	Antisense of CR1-F, ORF1 for strand-specific RT-PCR		GATCAAAGAACCCGAGATTCT
CR1-F-ORF2	Gene-specific qRT-PCR of CR1-F (ORF2) transposon	GAGGAGGACCAGGGAGAATC	CTCGTGAATTGTGGGAGGTT
F-ORF2-internal	Antisense of CR1-F, ORF2 for strand-specific RT-PCR		CCCTCAAGACAATCTGCTCCATAAT
CHICKEN DICER	Gene-specific qRT-PCR of Dicer	AGCAAGGCTGTTGAAGAGGA	GTTCTCTCCAGTCGCACACA
Dicer-internal	Antisense of Dicer primer		CTTCTCAAAGTTTTCAAAACCAGAA
L1-ORF1	Gene-specific qRT-PCR of LINE-1 (ORF1) family	TAAGGGCAGCCAGAGAGAAA	GCCTGGTGGTGACAAAATCT
L1-ORF1-internal	Antisense of L1, ORF1 for strand-specific RT-PCR		GGCATGATTTTGCAGCGGCTGGT
L1-ORF2	Gene-specific qRT-PCR of LINE-1 (ORF2) family	TCCAGCAGCACATCAAAAAG	CCAGTTTTTGCCCATTCAGT
L1-ORF2-internal	Antisense of LINE1, ORF2 for strand-specific RT-PCR		CAGGGACAATTTGACTTCCTCT
GENO-5' AND -3'	Retrotransposition assay	TTTATTGCCGATCCCCTCAGAAGAA	TCAAGGACGACGGCAACTACAAGA
CR1-B 5'UTR probes	5'-GCGGTCACAGCTCTCAGCCGGGCAGCTCTCATGAGAGCGGCTGACGCGGCGTGACATTGCCAGTGGCAA-3'		
(mixture of oligos used for	5'-GCTCTGTATCCATGCCCACCTGCCTAGAAAAAGCCTTTGAGAGGGCAGCGATGTGTCACTGCCCATCAGA-3'		
Northern Blot analysis)	5'-GACGGAGAGGAAGAGTCGGGGTGTGAGATCCACGTAGGCTGTTTTAACAAGTGTTACTGAGCCTGCTGGT-3'		

Primers for the consensus sequences of CR1-B transposons (Accession no. U88211), CR1-F transposons (Repbase Data submitted by Wicker el.al., 2005) and human LINE L1 elements (Accession no. AF148856) were designed according to Repbase (2005) and PubMed sequences. The Internal primers used for strand-specific RT-PCR (targeting the sense message of transposons) bind at least 100-200 bp downstram of gene-specific qRT-PCR primers. All the primers were designed by using Primer3 software.

Supplementary Table 2

5'UTR of CR1 elements in the chicken genome

No	chromosome	Strand	5' start	3' end	Length	Sub family
1	chr1	+	178129020	178129244	225	B
2	chr2	-	54618398	54618623	226	B
3	chr2	+	149637401	149637623	223	B
4	chr2	+	55496021	55496214	194	B
5	chr3	-	98157576	98157784	209	B
6	chrZ	+	71498907	71499286	380	B
7	chr2	-	51126275	51126431	157	B
8	chr5	-	5719610	5719735	126	B
9	chr1	+	97091047	97091460	414	C
10	chr4	-	23480114	23480558	445	C
11	chrZ	+	47797761	47798179	419	C
12	chr1	+	158140989	158141405	417	C
13	chr1	-	157153140	157153521	382	C
14	chr1	+	159677665	159678037	373	C
15	chr1	+	157391074	157391467	394	C
16	chr1	-	159316180	159316570	391	C
17	chrZ	+	26148926	26149338	413	C
18	chr1	+	165638455	165638797	343	C
19	chr1	+	76182436	76182792	357	C
20	chrZ	+	64048132	64048507	376	C
21	chr2	-	139474667	139475085	419	C
22	chr1	-	168538406	168538761	356	C
23	chr1	-	197718264	197718689	426	C
24	chr1	+	165902368	165902805	438	C
25	chr1	+	156485606	156485955	350	C
26	chr1	+	157842525	157842899	375	C
27	chr1	-	145880368	145880773	406	C
28	chr1	-	164917439	164917783	345	C
29	chr1	-	2598242	2599351	1110	D
30	chr2	+	117774539	117775645	1107	D
31	chr1	+	3486773	3487880	1108	D
32	chr2	+	150248459	150249529	1071	D
33	chr1	-	120116593	120117680	1088	D
34	chr1	+	116532535	116533637	1103	D
35	chr1	+	58900207	58901312	1106	D
36	chr6	-	10873710	10874795	1086	D
37	chr1	-	69170424	69171479	1056	D
38	chr1	-	86219179	86220296	1118	D
39	chr3	-	103773050	103774127	1078	D
40	chr1	+	86318315	86319423	1109	D
41	chrW	+	45083	46187	1105	D
42	chr1	-	84126453	84127564	1112	D
43	chr1	-	28394565	28395595	1031	D
44	chr2	+	130931930	130933027	1098	D
45	chr2	+	61192399	61193519	1121	D
46	chr1	-	170681165	170682259	1095	D
47	chr2	-	150100100	150101172	1073	D
48	chr1	-	123655270	123656363	1094	D
49	chr3	-	98980578	98981675	1098	D
50	chr3	-	62481463	62482519	1057	D
51	chr4	+	85719439	85720441	1003	D

52	chr6	-	16897637	16898637	1001	D
53	chr1	-	5999193	6000200	1008	D
54	chr1	-	130083443	130084525	1083	D
55	chr2	-	142717034	142717970	937	D
56	chr3	-	87228986	87230037	1052	D
57	chr1	-	43065337	43066424	1088	D
58	chr2	-	47556550	47557648	1099	D
59	chr4	-	36754733	36756266	1534	D
60	chr2	+	13514709	13515737	1029	D
61	chr2	+	66920339	66921436	1098	D
62	chrZ	-	40496340	40497439	1100	D
63	chr6	-	8109891	8110982	1092	D
64	chr1	+	55107540	55108637	1098	D
65	chr3	+	72328315	72329376	1062	D
66	chr1	+	129224227	129225294	1068	D
67	chrZ	-	21102645	21103750	1106	D
68	chr1	-	13246926	13248020	1095	D
69	chr2	-	146059612	146068012	8401	D
70	chr2	+	42112941	42113921	981	D
71	chr2	+	91023808	91024922	1115	D
72	chr1	+	161993915	161994893	979	D
73	chr1	+	19340818	19341814	997	D
74	chr2	+	96578739	96579849	1111	D
75	chr4	+	80946959	80948060	1102	D
76	chr7	-	7264827	7265829	1003	D
77	chr3	+	82694524	82695563	1040	D
78	chr4	+	56573176	56574203	1028	D
79	chr2	+	51141830	51142932	1103	D
80	chrZ	+	15844273	15845256	984	D
81	chr3	+	33679717	33680808	1092	D
82	chr1	-	32705549	32706455	907	D
83	chr3	-	26768089	26769021	933	D
84	chr1	-	172794310	172795372	1063	D
85	chr2	-	138829288	138830542	1255	D
86	chr4	+	58854955	58855872	918	D
87	chr1	-	177616726	177617757	1032	D
88	chrZ	+	39223898	39225005	1108	D
89	chr2	-	118945288	118946225	938	D
90	chr1	-	140403808	140404757	950	D
91	chr1	+	119659411	119660463	1053	D
92	chr5	-	54630992	54632052	1061	D
93	chr1	-	197698255	197699165	911	D
94	chr4	-	45230610	45231713	1104	D
95	chr2	+	34751455	34752373	919	D
96	chr4	-	58068733	58069603	871	D
97	chr3	-	37338559	37339550	992	D
98	chr8	+	25011897	25012993	1097	D
99	chrZ	-	74206765	74207796	1032	D
100	chr2	-	56344223	56345086	864	D
101	chr3	-	67670121	67671025	905	D
102	chr2	+	137607965	137608844	880	D
103	chr1	-	5242047	5243148	1102	D
104	chr6	+	2161469	2162179	711	F
105	chr1	-	166388213	166388712	500	F
106	chr1	+	73044218	73045218	1000	F
107	chr1	-	166383675	166386713	1000	F
108	chr3	-	76930060	76933041	1000	F
109	chr5	+	2882232	2883232	1000	F

110	chr1	+	162316710	162317710	1000	F
111	chr1	+	27442522	27442901	380	G
112	chr3	-	64637470	64637830	361	G
113	chr2	+	72615140	72615518	379	G
114	chr3	-	82602312	82602670	359	G
115	chr1	+	161842967	161843342	376	G
116	chr1	-	163889547	163889906	360	G
117	chr1	-	102633640	102634013	374	G
118	chr2	-	69511421	69511775	355	G
119	chr1	-	159230221	159230599	379	G
120	chr1	+	159919317	159919692	376	G
121	chr1	-	170097865	170098243	379	G
122	chr1	-	163904265	163904613	349	G
123	chr1	+	76517136	76517514	379	G
124	chr1	+	168746897	168747256	360	G
125	chr1	-	155664218	155664574	357	G
126	chr1	+	155748497	155748856	360	G
127	chr3	-	81890687	81891034	348	G
128	chr1	-	153300977	153301332	356	G
129	chr1	+	76059160	76059537	378	G
130	chr1	-	156702193	156702552	360	G
131	chr1	-	33943511	33943875	365	G
132	chr1	+	129999192	129999545	354	G
133	chr2	+	37211163	37211516	354	G
134	chr3	+	55421482	55421848	367	G
135	chr3	-	19260507	19260880	374	G
136	chr2	+	93540925	93541284	360	G
137	chr1	+	75562783	75563141	359	G
138	chr1	+	145665104	145665468	365	G
139	chrZ	+	56388386	56388730	345	G
140	chr4	+	19147751	19148116	366	G
141	chr1	+	103274130	103274473	344	G
142	chr2	+	50490057	50490393	337	G
143	chr4	-	74509280	74509640	361	G
144	chr5	-	57504259	57504607	349	G
145	chr4	-	43214314	43214662	349	G
146	chr2	-	84675749	84676080	332	G
147	chrZ	+	5153302	5153667	366	G
148	chr1	+	167283013	167283343	331	G
149	chr3	+	28742966	28743329	364	G
150	chr1	-	59852381	59852662	282	G
151	chr3	+	11443751	11444079	329	G
152	chrZ	+	30435788	30436113	326	G
153	chr4	+	33870962	33871298	337	G
154	chr2	+	54835577	54835862	286	G
155	chr1	-	154784640	154784958	319	G
156	chr1	+	157032084	157032413	330	G
157	chr2	-	54944812	54945090	279	G
158	chr1	-	98198559	98199117	559	G
159	chr1	+	39660808	39661098	291	G
160	chr3	-	25742070	25742409	340	G
161	chr2	+	75127413	75127791	379	G
162	chr1	+	152100352	152101062	711	H
163	chr1	-	167640848	167641487	640	H
164	chr1	+	162076568	162077245	678	H
165	chr1	-	163086856	163087498	643	H
166	chr1	-	159541089	159541687	599	H

167	chr4	-	29364401	29364978	578	H
168	chr1	-	167908970	167909518	549	H
169	chr1	-	157915170	157915755	586	H

Supplementary Table 3: The 5'UTR sequences of the chicken CR1 elements. The highly conserved regions in the 5'UTR of CR1 subfamily are shaded. Dots indicate identical nucleotides. Dash represents the truncation of nucleotides. The chromosome and position of nucleotides are shown in left.

CR1-B Subfamily: CLUSTALX (1.83) Multiple Sequence Alignment

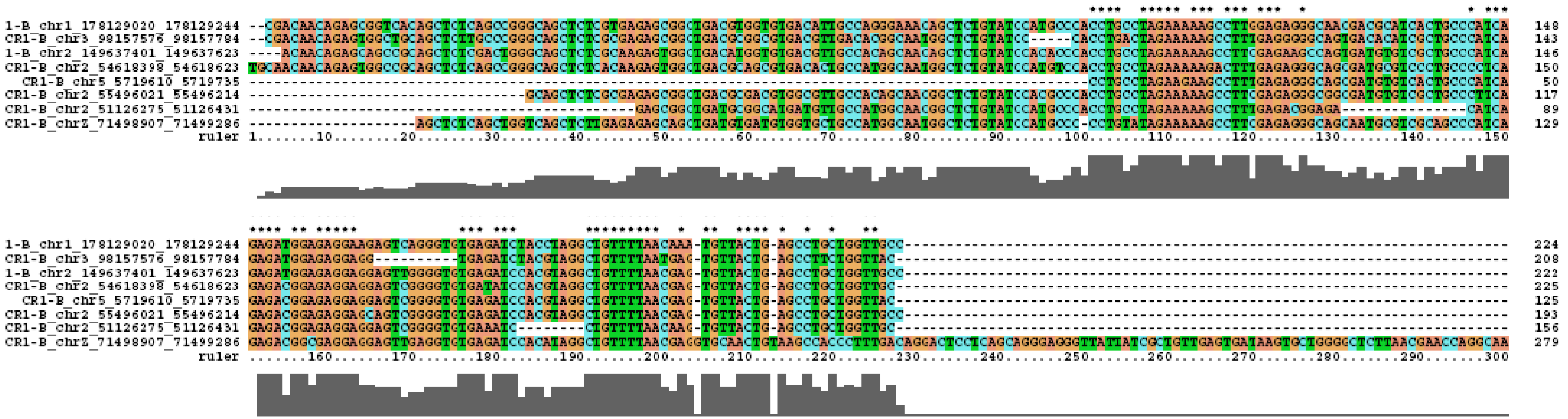

CR1-F Subfamily: CLUSTALX (1.83) Multiple Sequence Alignment

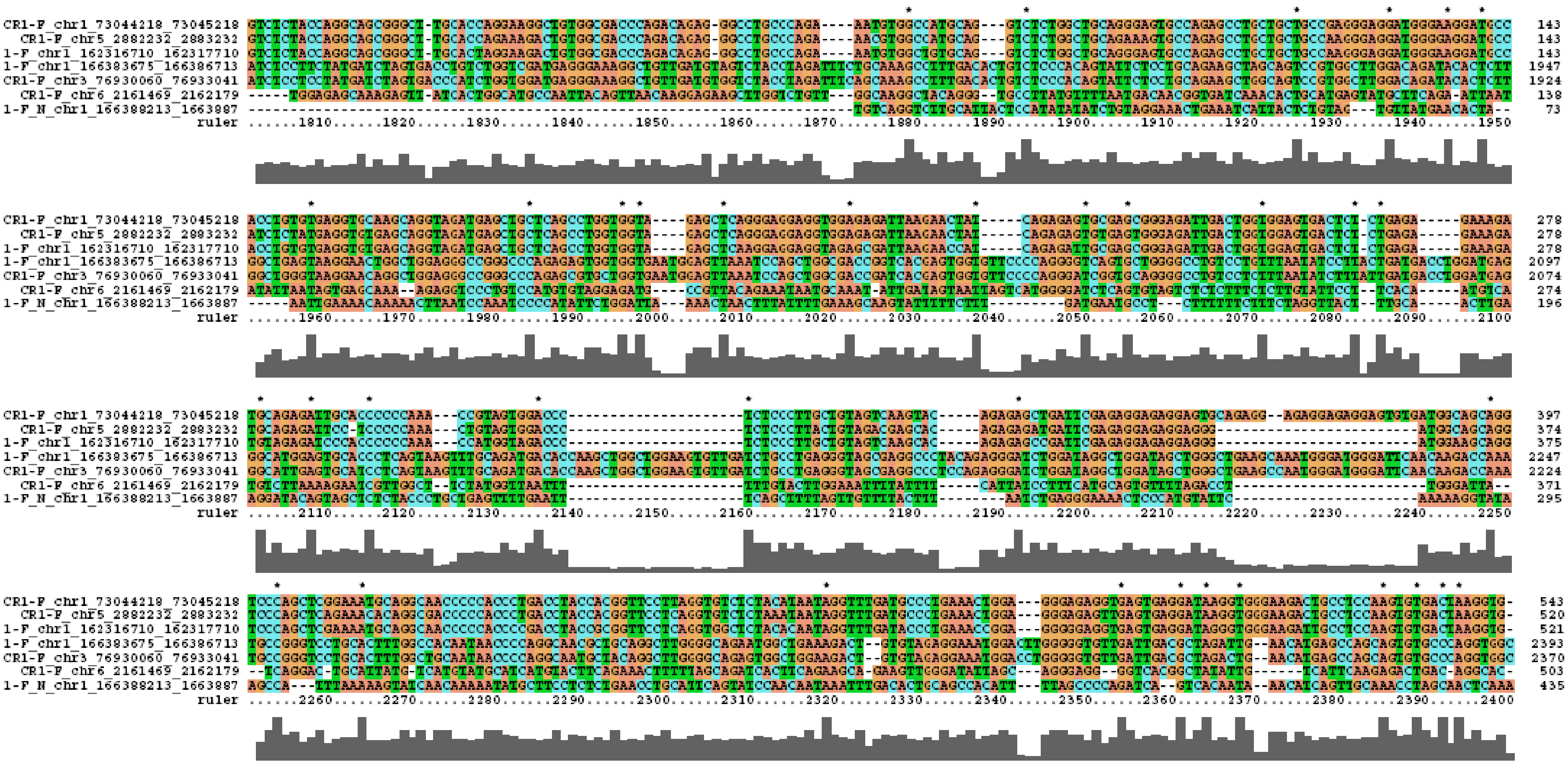

CR1-F Subfamily: CLUSTALX (1.83) Multiple Sequence Alignment

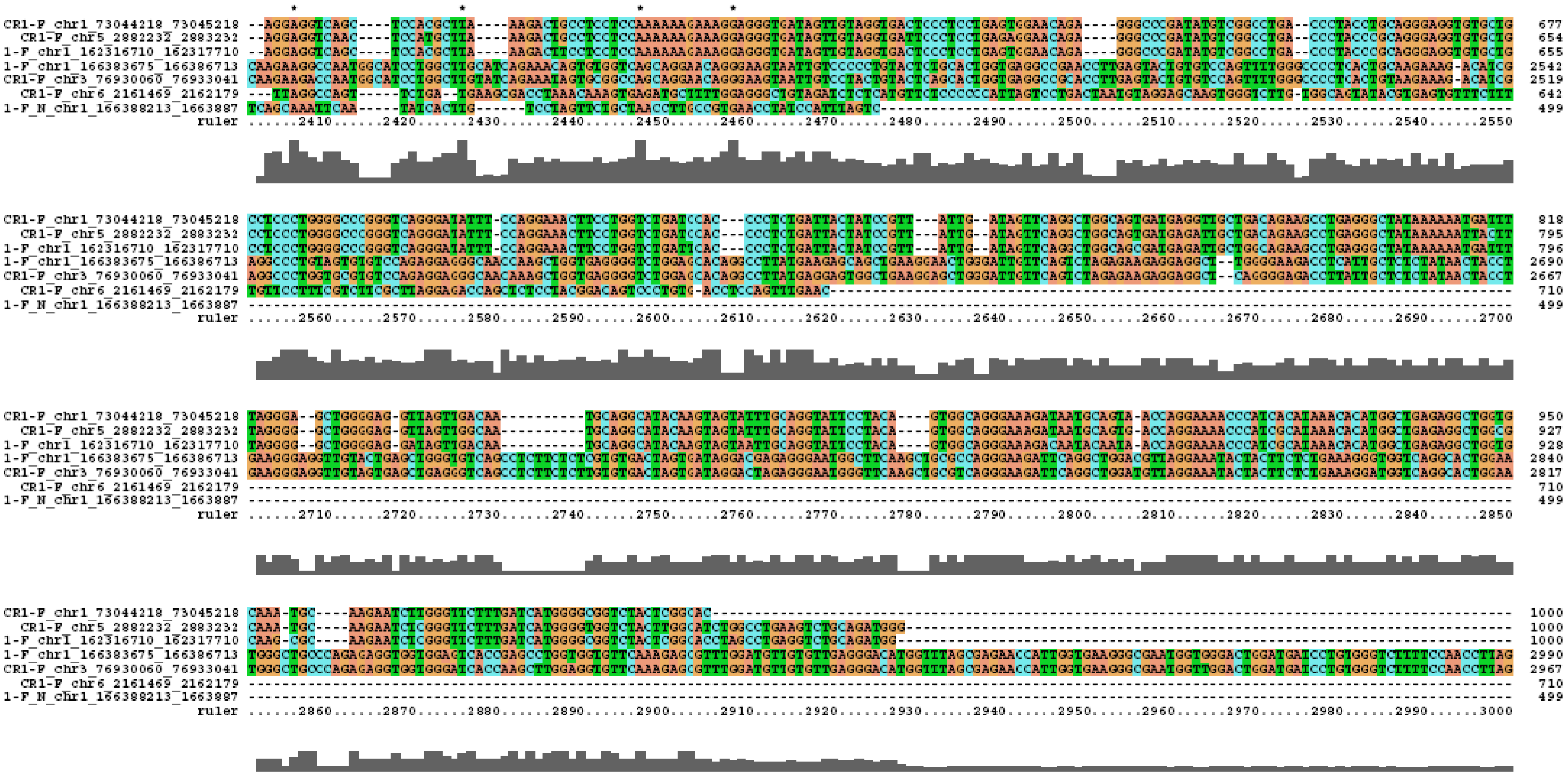

CR1-C Subfamily: CLUSTALX (1.83) Multiple Sequence Alignment

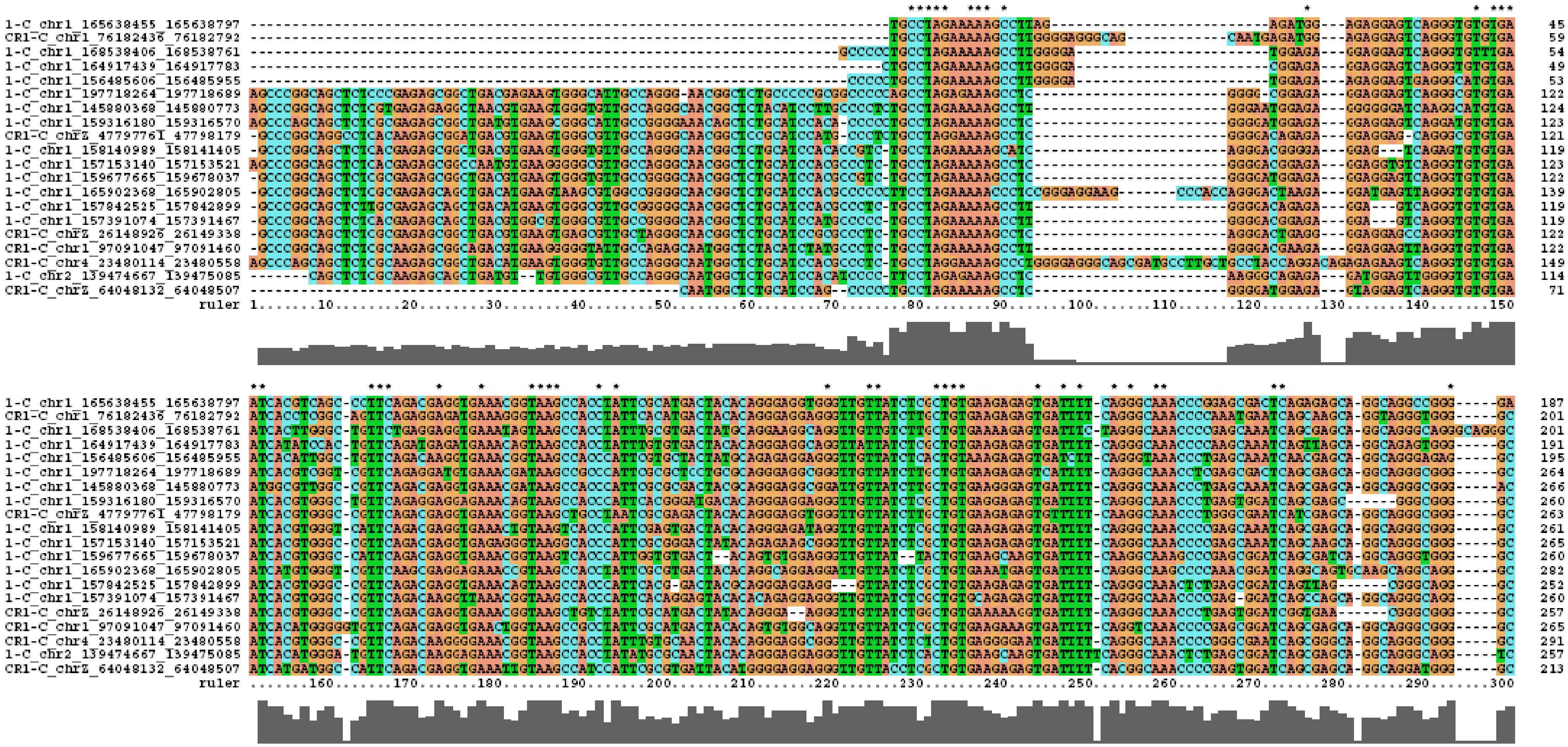

CR1-C Subfamily: CLUSTALX (1.83) Multiple Sequence Alignment

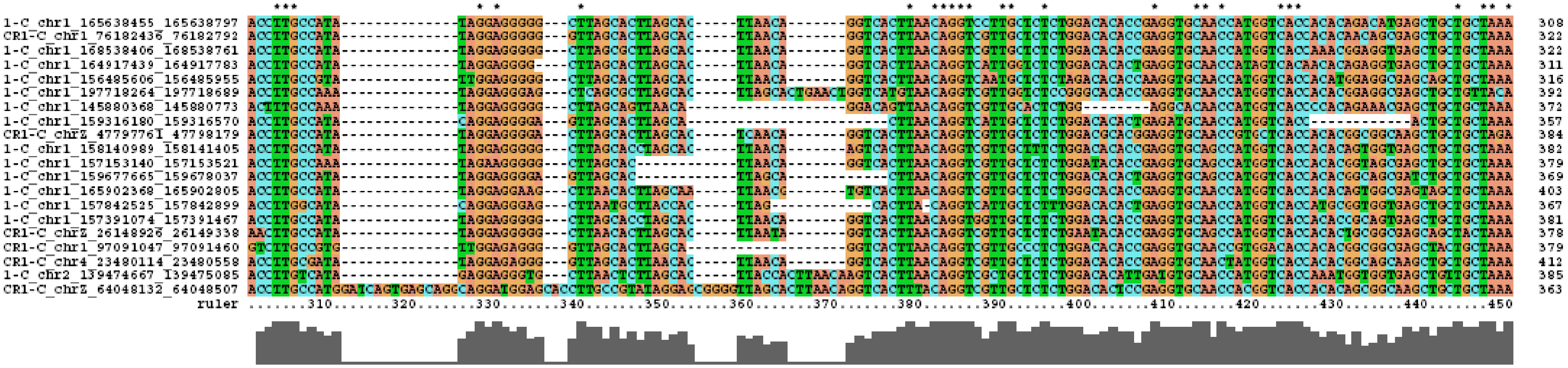

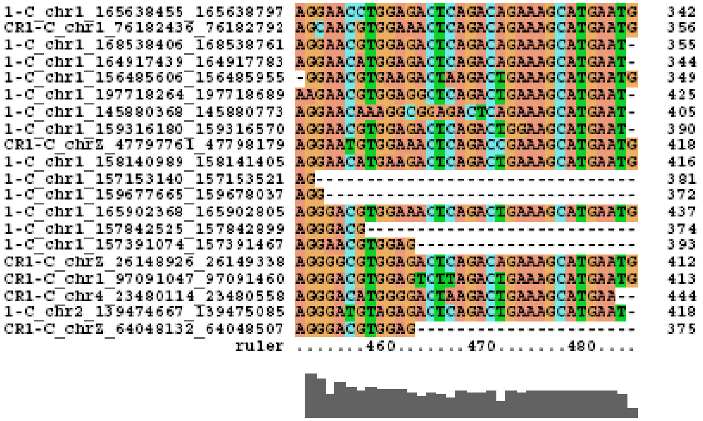

CR1-D Subfamily: CLUSTALX (1.83) Multiple Sequence Alignment

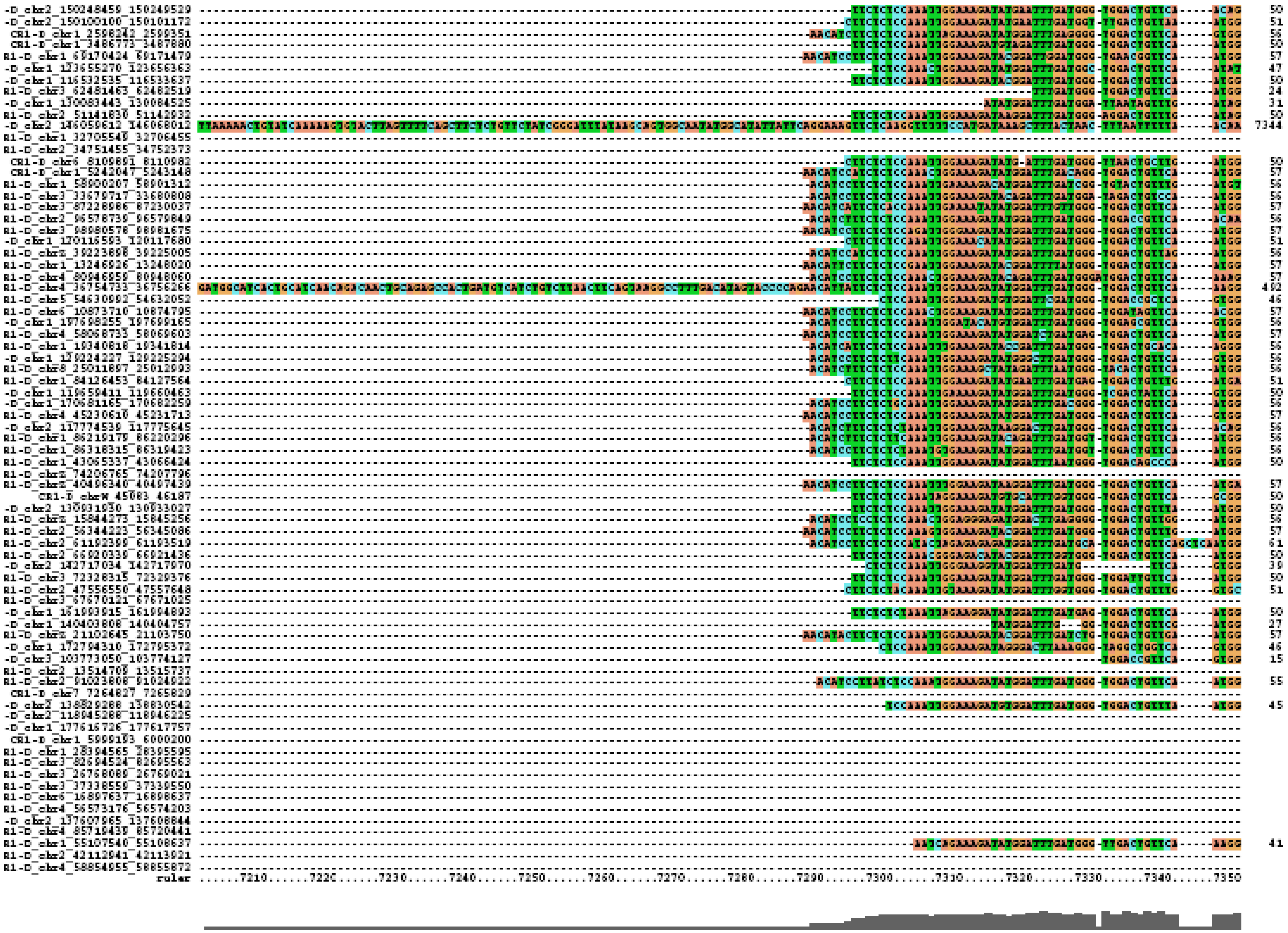

CR1-D Subfamily: CLUSTALX (1.83) Multiple Sequence Alignment

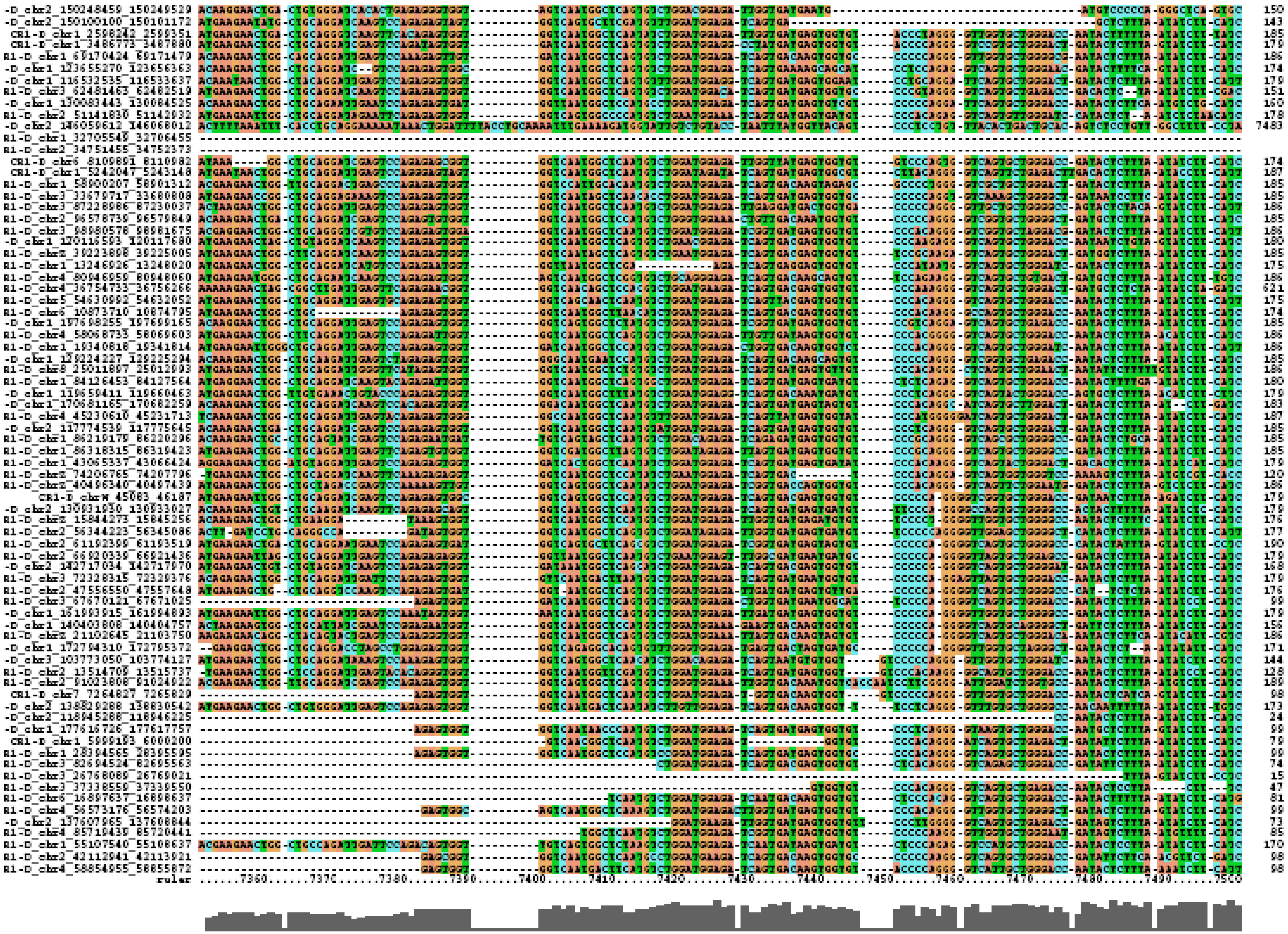

CR1-D Subfamily: CLUSTALX (1.83) Multiple Sequence Alignment

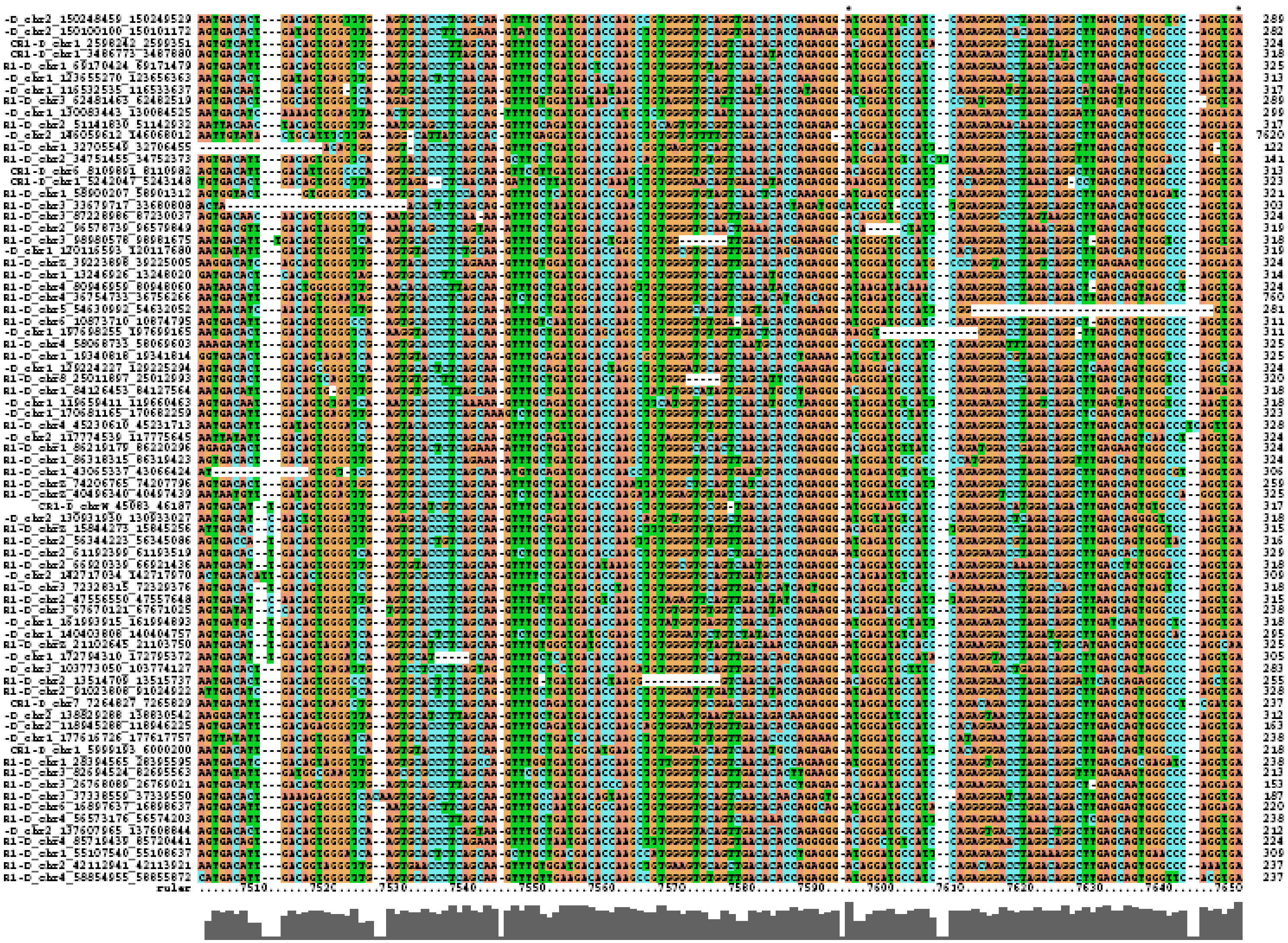

CR1-D Subfamily: CLUSTALX (1.83) Multiple Sequence Alignment

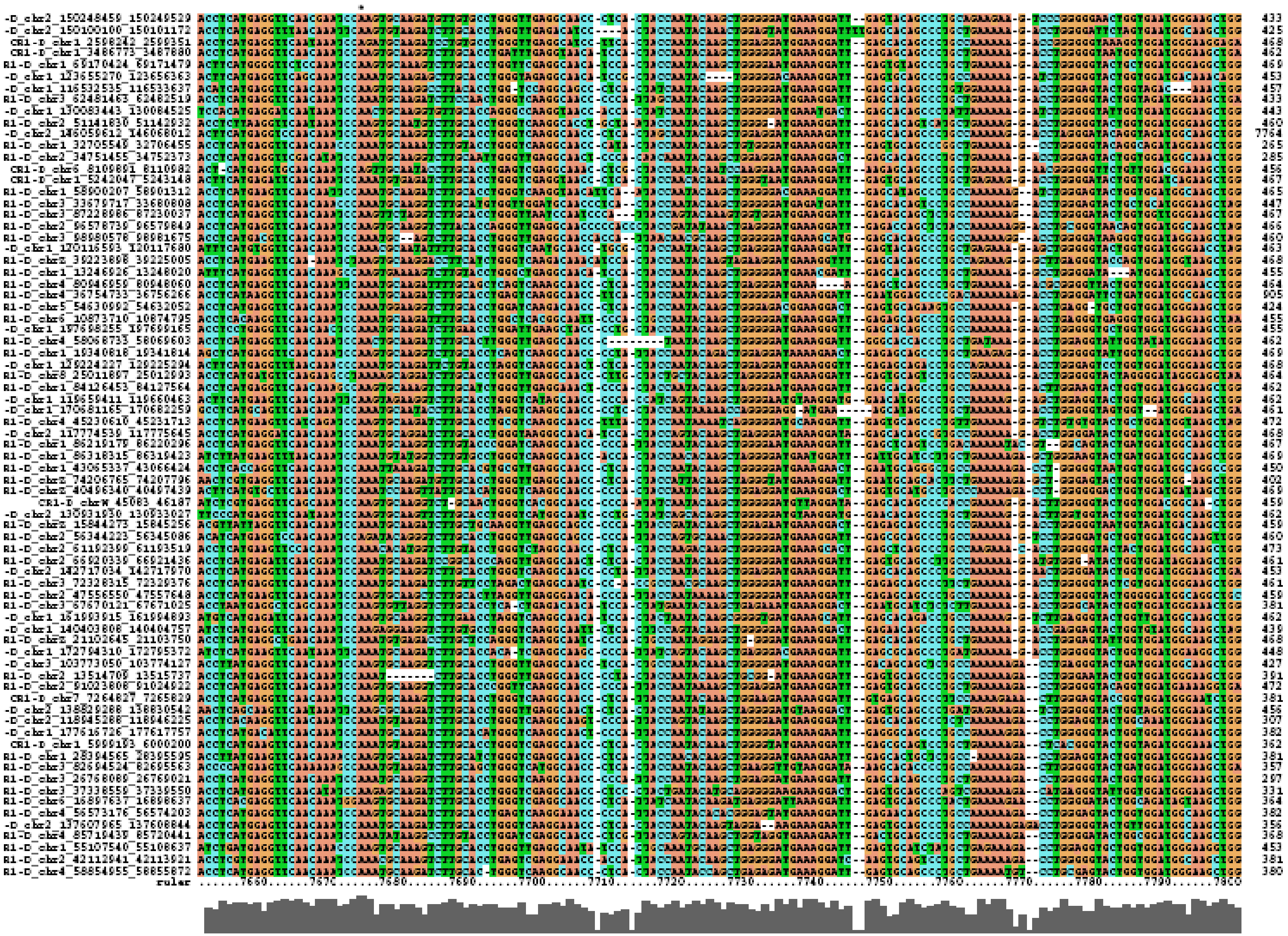

CR1-D Subfamily: CLUSTALX (1.83) Multiple Sequence Alignment

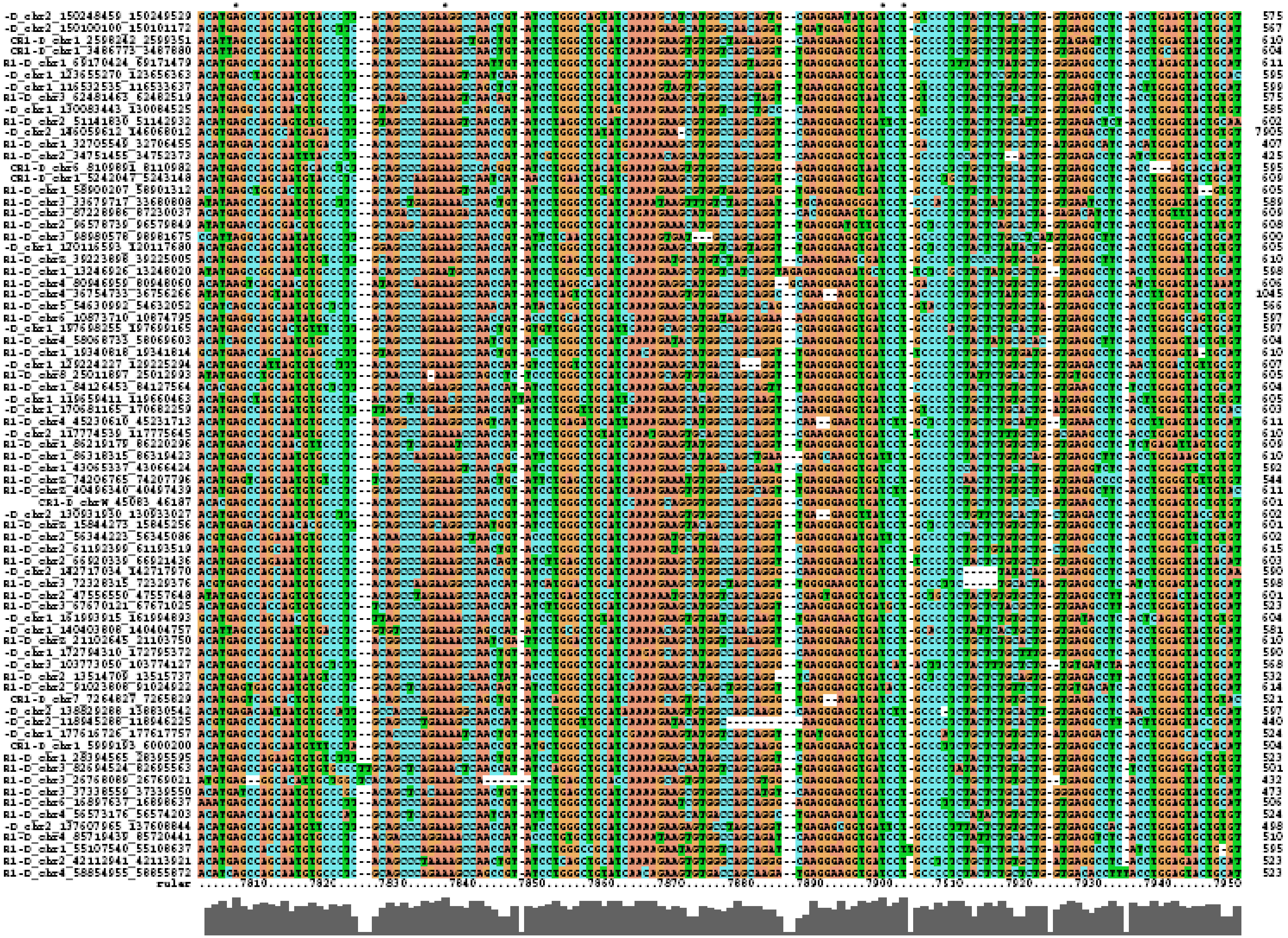

CR1-D Subfamily: CLUSTALX (1.83) Multiple Sequence Alignment

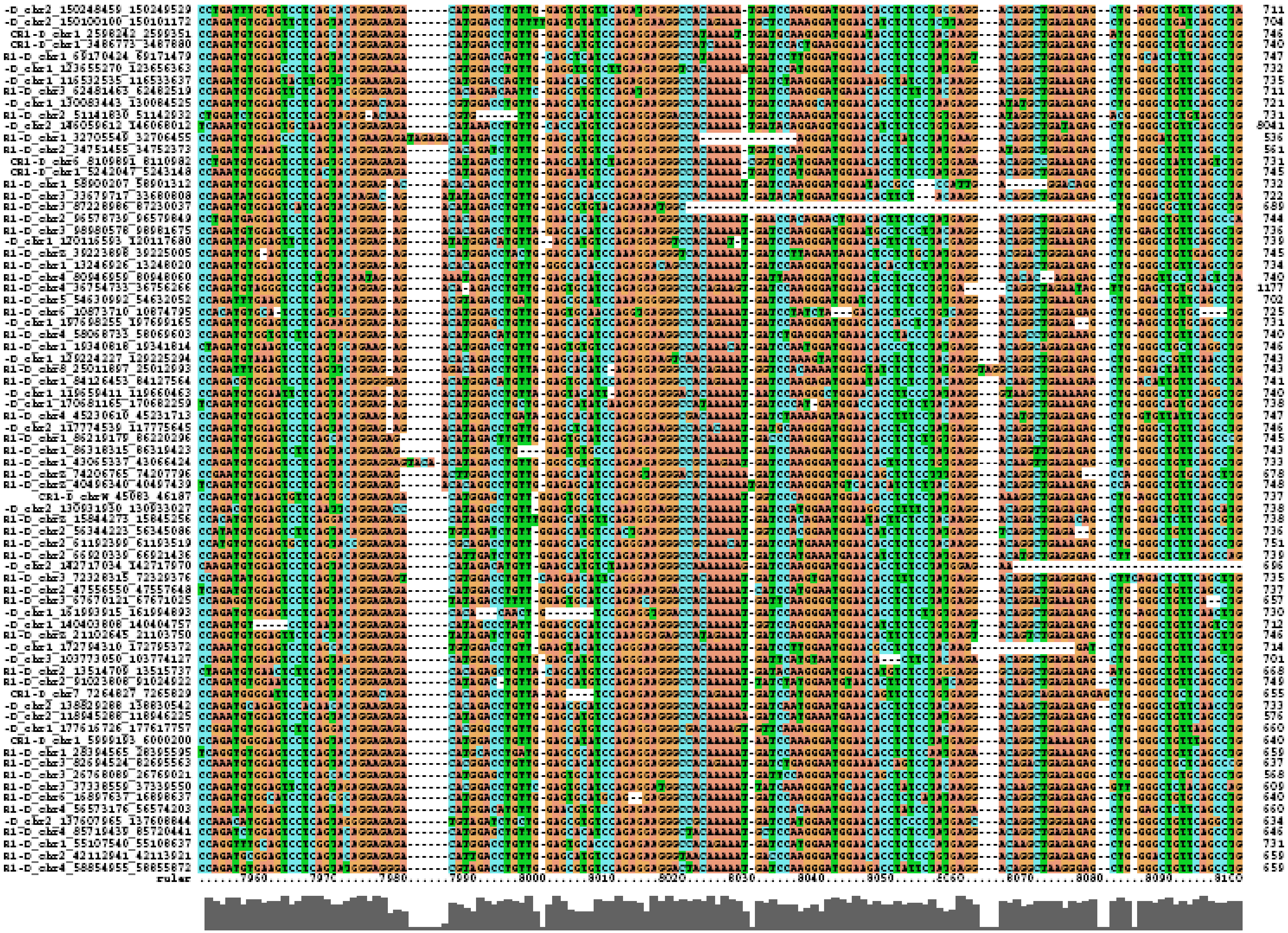

CR1-D Subfamily: CLUSTALX (1.83) Multiple Sequence Alignment

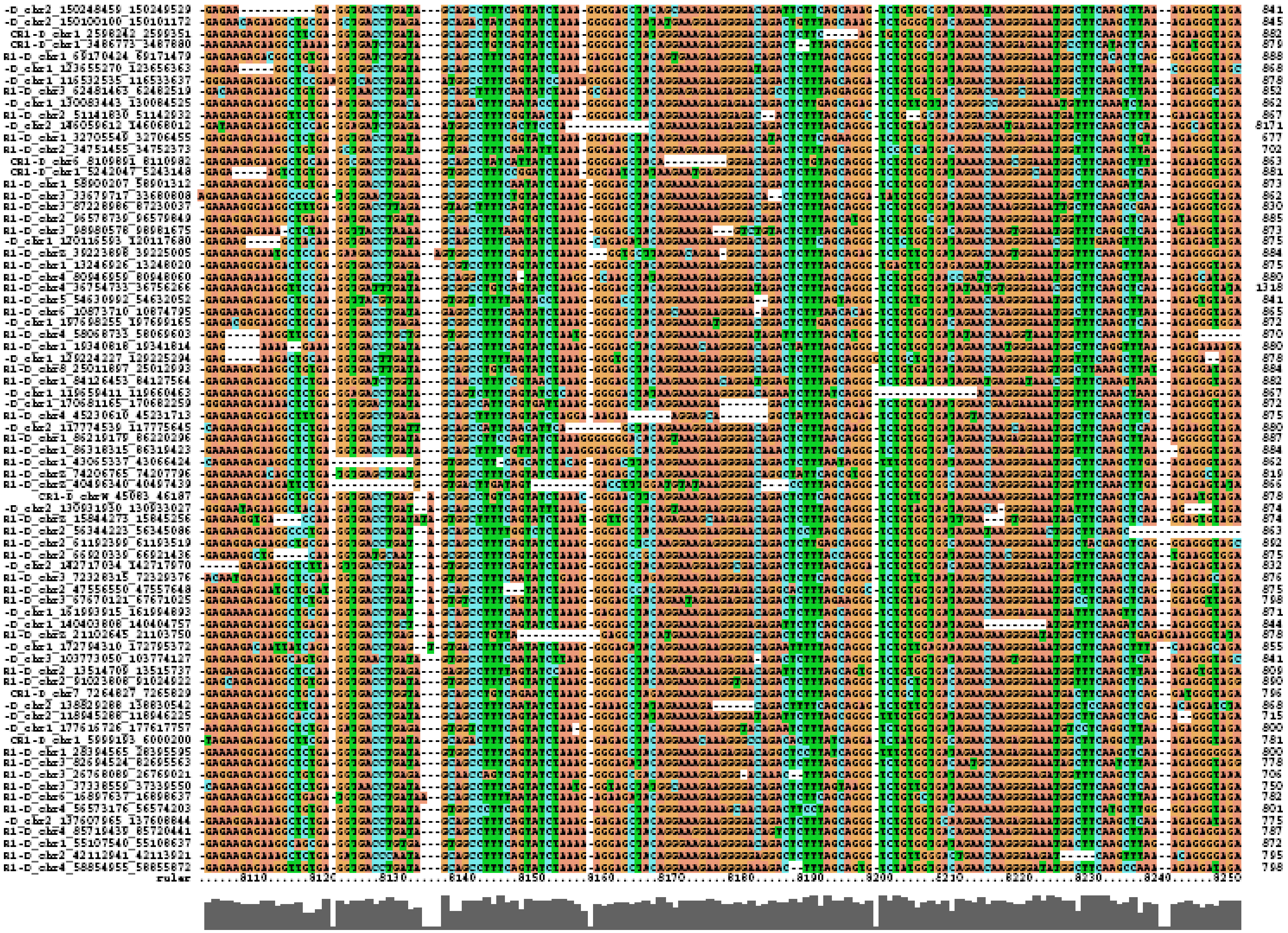

CR1-D Subfamily: CLUSTALX (1.83) Multiple Sequence Alignment

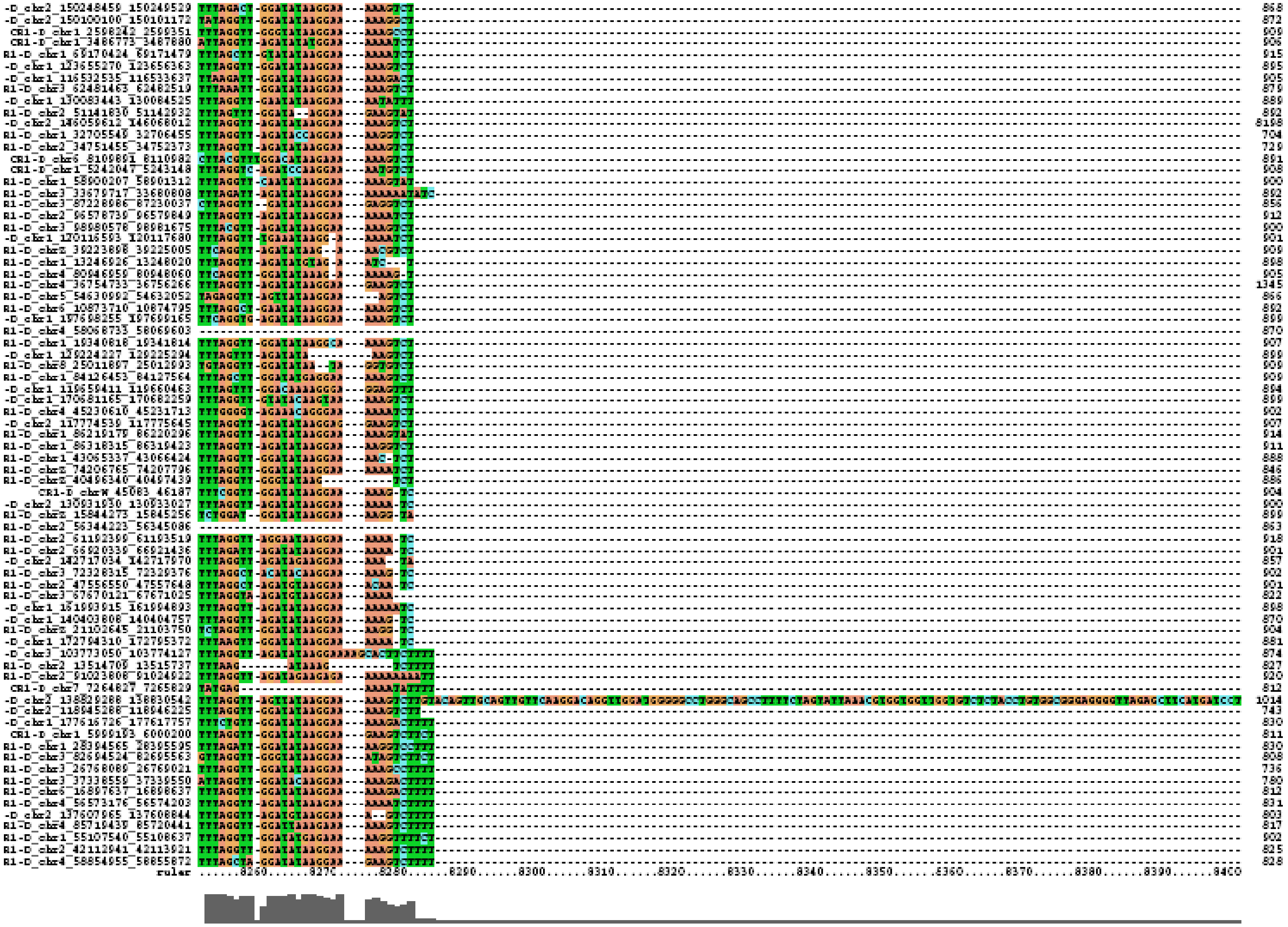

CR1-G Subfamily: CLUSTALX (1.83) Multiple Sequence Alignment

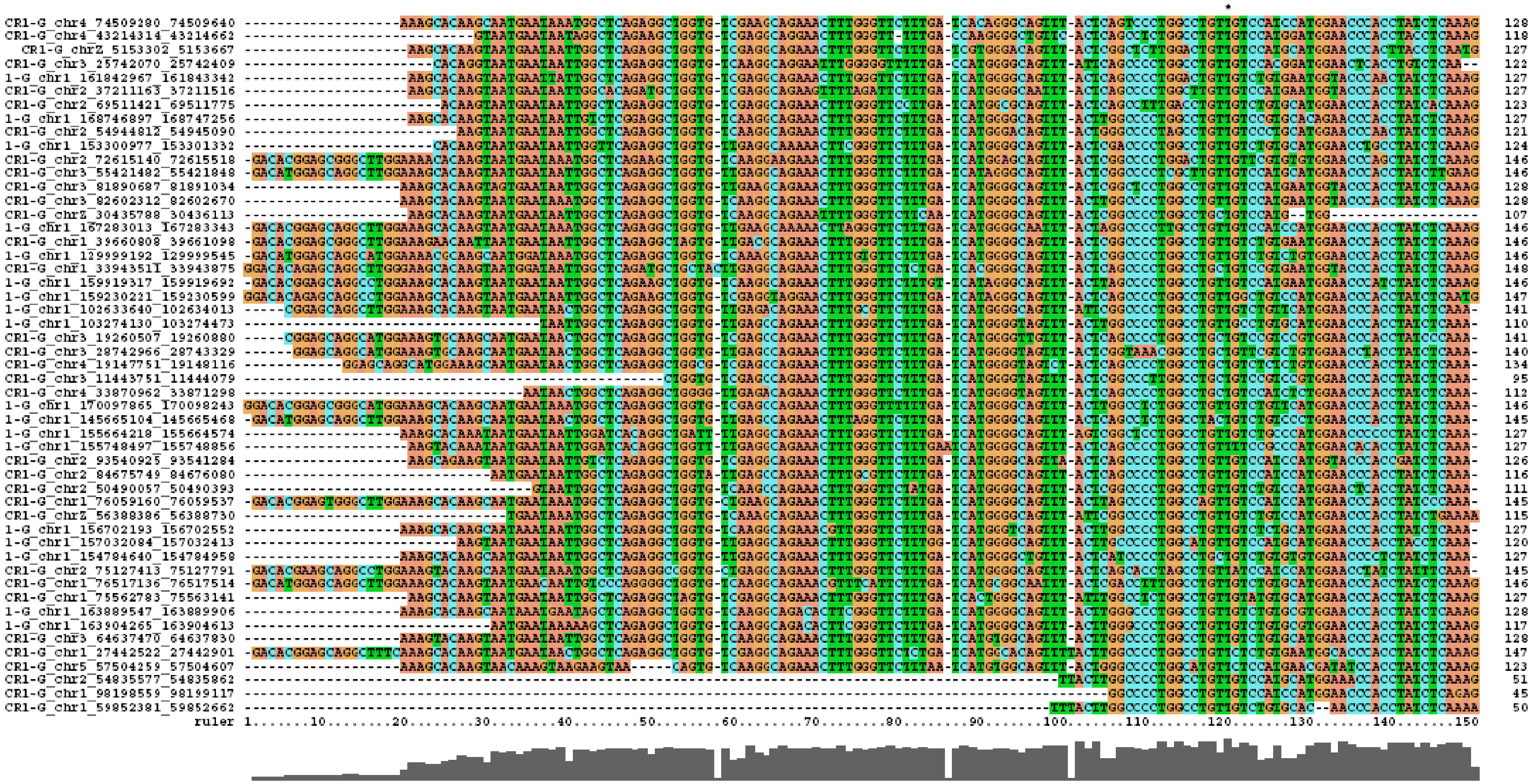

CR1-G Subfamily: CLUSTALX (1.83) Multiple Sequence Alignment

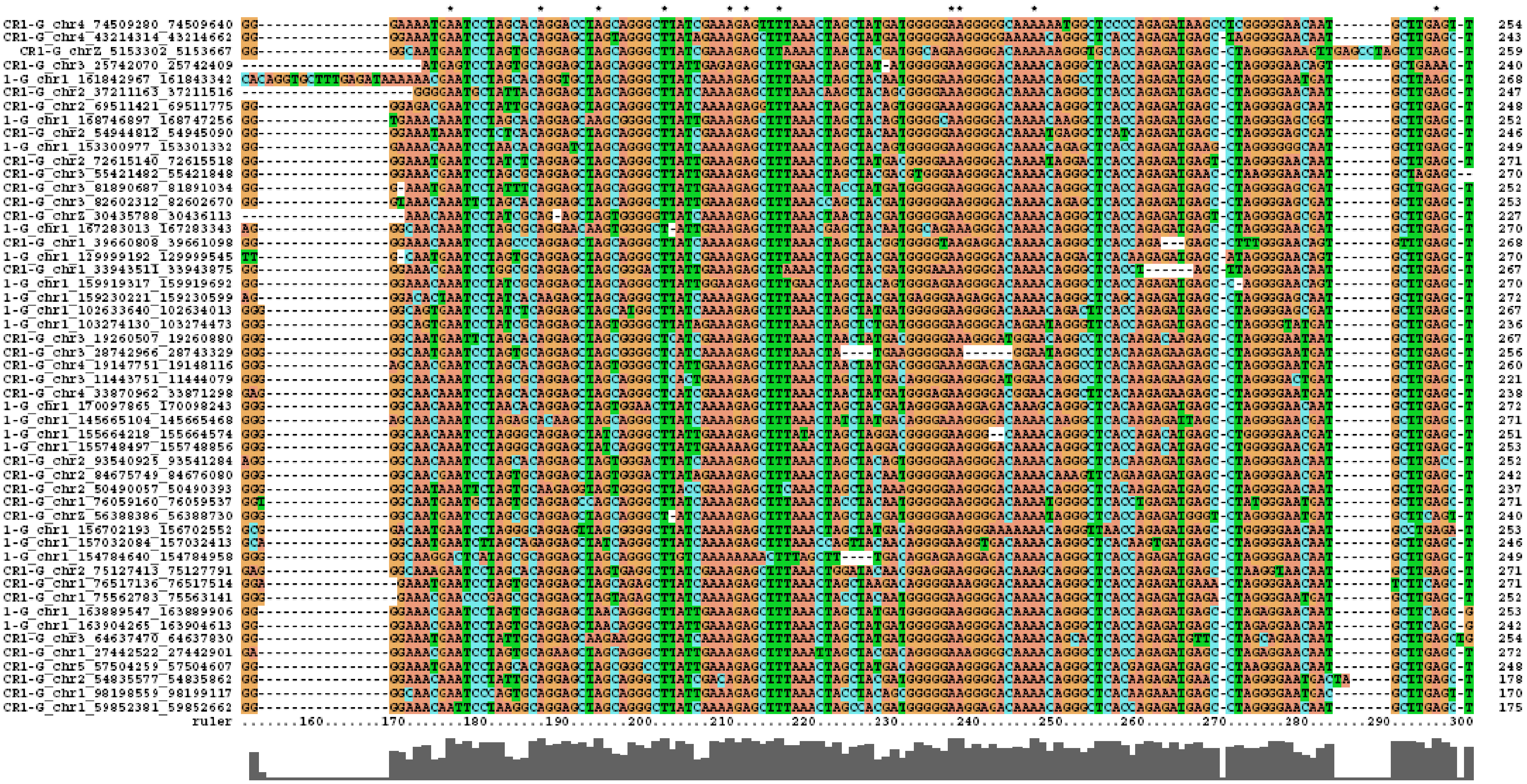

CR1-G Subfamily: CLUSTALX (1.83) Multiple Sequence Alignment

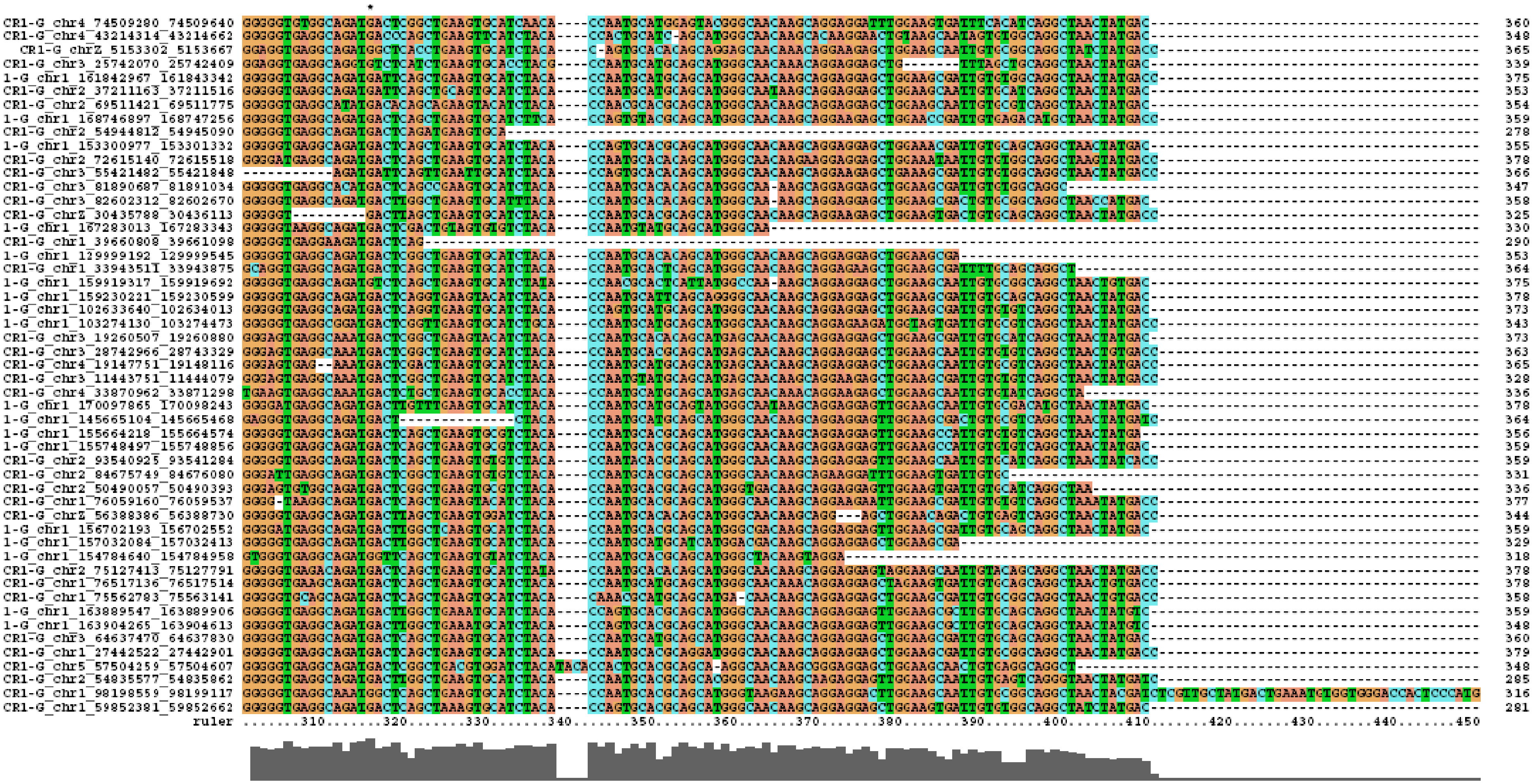

CR1-H Subfamily: CLUSTALX (1.83) Multiple Sequence Alignment

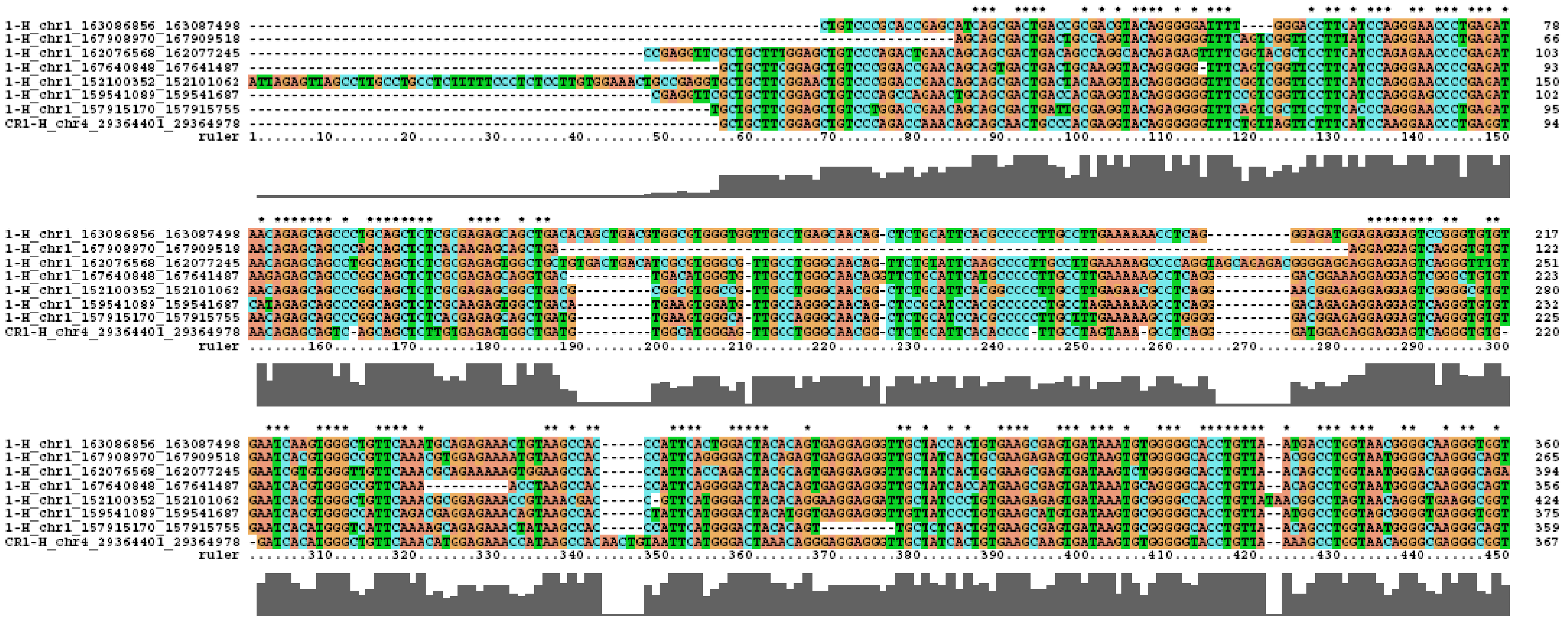

CR1-H Subfamily: CLUSTALX (1.83) Multiple Sequence Alignment

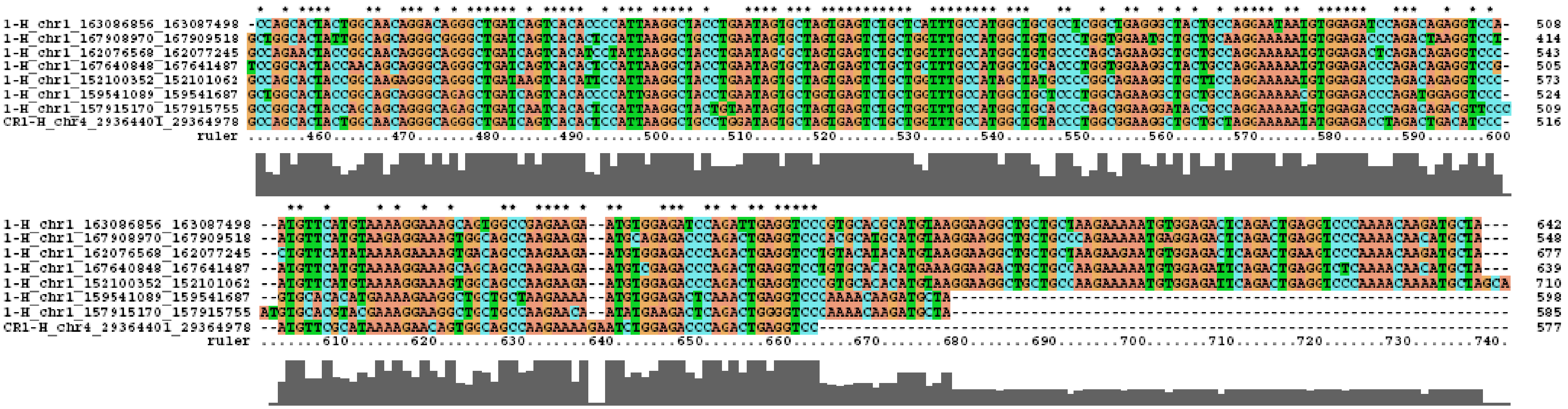

Supplementary Table 4: Transcription factor binding sites in chicken CR1 subfamilies

CR1-C subfamily   Graph version

Matrix identifier	Position (strand)	Core match	Matrix match	Sequence (always the (+)-strand is shown)	Factor name
V$GKLF_01	79 (-)	0.87	0.58	CCTAGnnaaancnn	GKLF
V$HOXA3_01	79 (+)	0.971	0.753	CCTAGnnaa	HOXA3
V$CDXA_01	83 (-)	0.882	0.819	gnNAAAN	CdxA
V$OCT1_04	138 (-)	0.877	0.677	nnnnnnnntnTGAATnnnnnnnn	Oct-01
V$CEBP_01	144 (+)	0.885	0.695	nnTNTGAatnnnn	C/EBP
V$MSX1_01	145 (+)	0.873	0.567	ntnTGAATn	Msx-1
V$CDXA_01	146 (-)	0.828	0.783	tnTGAAT	CdxA
V$HOXA3_01	179 (-)	0.92	0.69	nnnnNTAAG	HOXA3
V$OCT1_03	180 (+)	0.9	0.825	nnnNTAAGnnnnc	Oct-01
V$CDXA_01	181 (-)	0.79	0.72	nnNTAAG	CdxA
V$GKLF_01	372 (+)	0.852	0.551	nnnnnnntnNCAGG	GKLF
V$USF_02	378 (+)	0.935	0.645	ntnnCAGGTnnntg	USF
V$CEBP_01	386 (+)	0.759	0.633	tnNNTGNnntnnn	C/EBP

CR1-D subfamily   Graph version

Matrix identifier	Position (strand)	Core match	Matrix match	Sequence (always the (+)-strand is shown)	Factor name
V$GKLF_01	7411 (+)	0.798	0.571	cnnnnncngGNNGG	GKLF
V$GKLF_01	7458 (+)	0.81	0.621	ggngnnngnGNTGG	GKLF
V$GKLF_01	7459 (+)	0.891	0.665	gngnnngngNTGGG	GKLF
V$GKLF_01	7507 (+)	0.813	0.54	nnnnnngnnNGNGG	GKLF
V$GKLF_01	7508 (+)	0.906	0.608	nnnnngnnnGNGGG	GKLF
V$GKLF_01	7556 (+)	0.813	0.583	ncnnnnngnNGNGG	GKLF
V$GKLF_01	7557 (+)	0.906	0.614	cnnnnngnnGNGGG	GKLF
V$GKLF_01	7558 (+)	0.92	0.662	nnnnngnngNGGGG	GKLF
V$MZF1_02	7562 (+)	0.805	0.697	ngnngNGGGGngn	MZF1
V$MZF1_01	7565 (+)	0.843	0.832	ngnGGGGN	MZF1
V$GKLF_01	7579 (+)	0.906	0.592	nncnnnnnnGNGGG	GKLF
V$GKLF_01	7585 (+)	0.772	0.652	nnngngggnANNGG	GKLF

V$GKLF_01	7601 (+)	0.813	0.563	gccnnnnncNGNGG	GKLF
V$GKLF_01	7602 (+)	0.906	0.604	ccnnnnncnGNGGG	GKLF
V$GKLF_01	7602 (-)	0.772	0.55	CCNNNnncngnggg	GKLF
V$GKLF_01	7613 (+)	0.777	0.578	gggnnnnngNCNGG	GKLF
V$GKLF_01	7625 (+)	0.813	0.667	ggcnngagnNGNGG	GKLF
V$GKLF_01	7626 (+)	0.906	0.707	gcnngagnnGNGGG	GKLF
V$GKLF_01	7634 (+)	0.772	0.584	ngngggcnnNNNGG	GKLF
V$GKLF_01	7682 (+)	0.772	0.533	gnnnnngcnNNNGG	GKLF
V$GKLF_01	7683 (+)	0.879	0.596	nnnnngcnnNNGGG	GKLF
V$GKLF_01	7689 (+)	0.772	0.604	cnnnngggnNNNGG	GKLF
V$GKLF_01	7728 (+)	0.772	0.59	nggngnnngNNNGG	GKLF
V$GKLF_01	7764 (+)	0.879	0.565	nnnnnnnnnNNGGG	GKLF
V$GKLF_01	7772 (+)	0.772	0.567	nnngggnnnNNNGG	GKLF
V$GKLF_01	7775 (+)	0.839	0.577	gggnnnnnnGGNGG	GKLF
V$GKLF_01	7779 (+)	0.798	0.599	nnnnnggngGNNGG	GKLF
V$GKLF_01	7787 (+)	0.798	0.597	ggnnggnnnGNNGG	GKLF
V$GKLF_01	7842 (+)	0.891	0.581	ncnnnnnnnNTGGG	GKLF
V$GKLF_01	7862 (+)	0.772	0.561	nnnnngnngNNNGG	GKLF
V$GKLF_01	7870 (+)	0.798	0.586	gnnnggnnnGNNGG	GKLF
V$GKLF_01	7878 (+)	0.772	0.561	ngnnggnnnNNNGG	GKLF
V$GKLF_01	7879 (+)	0.879	0.634	gnnggnnnnNNGGG	GKLF
V$GKLF_01	7882 (+)	0.839	0.611	ggnnnnnngGGNGG	GKLF
V$GC_01	7887 (+)	0.935	0.803	nnngGGNGGngntn	GC box
V$SP1_Q6	7887 (+)	0.904	0.808	nnngGGNGGngnt	Sp1
V$GKLF_01	7905 (-)	0.813	0.596	CCNCNcnncnnngn	GKLF
V$GKLF_01	7927 (+)	0.788	0.531	ngnccncnnCCNGG	GKLF
V$GKLF_01	7930 (-)	0.813	0.615	CCNCNnccnggngn	GKLF
V$GKLF_01	7947 (+)	0.813	0.61	gnnnccngnNGNGG	GKLF
V$GKLF_01	7962 (+)	0.772	0.577	gnnctcngnNNNGG	GKLF
V$GKLF_01	8005 (+)	0.798	0.572	nnnnncngnGNNGG	GKLF
V$GKLF_01	8006 (+)	0.879	0.639	nnnncngngNNGGG	GKLF
V$GKLF_01	8031 (+)	0.772	0.564	gnnncnnngNNNGG	GKLF
V$GKLF_01	8057 (+)	0.777	0.553	nnnnggnnnNCNGG	GKLF
V$GKLF_01	8074 (+)	0.813	0.562	nnngngnnnNGNGG	GKLF
V$GKLF_01	8075 (+)	0.906	0.625	nngngnnnnGNGGG	GKLF
V$GKLF_01	8100 (+)	0.798	0.627	gngngnngnGNNGG	GKLF
V$GKLF_01	8109 (+)	0.772	0.567	gnnggcnnnNNNGG	GKLF

V$GKLF_01	8147 (+)	0.879	0.575	nnnncnnnnNNGGG	GKLF
V$GKLF_01	8164 (+)	0.798	0.542	nnnngnnnnGNNGG	GKLF
V$GKLF_01	8165 (+)	0.879	0.626	nnngnnnngNNGGG	GKLF
V$GKLF_01	8166 (+)	0.92	0.658	nngnnnngnNGGGG	GKLF
V$GKLF_01	8183 (+)	0.798	0.572	gnnnnnnnaGNNGG	GKLF
V$GKLF_01	8184 (+)	0.879	0.665	nnnnnnnagNNGGG	GKLF
V$GKLF_01	8205 (+)	0.772	0.54	gngnnngnnNNNGG	GKLF
V$GKLF_01	8206 (+)	0.879	0.601	ngnnngnnnNNGGG	GKLF
V$GKLF_01	8207 (+)	0.92	0.644	gnnngnnnnNGGGG	GKLF
V$GKLF_01	8213 (+)	0.772	0.626	nnnnggggnNNNGG	GKLF
V$GKLF_01	8243 (+)	0.772	0.54	ngngnngnnNNNGG	GKLF
V$GKLF_01	8256 (+)	0.772	0.535	gnnnngnnnNNNGG	GKLF
V$GKLF_01	8441 (+)	0.813	0.617	nnnngngngNGNGG	GKLF
V$GKLF_01	8446 (+)	0.813	0.673	ngngngnggNGNGG	GKLF
V$GKLF_01	8516 (+)	0.772	0.539	ggngnnnnnNNNGG	GKLF

CR1-G subfamily   Graph version

Matrix identifier	Position (strand)	Core match	Matrix match	Sequence (always the (+)-strand is shown)	Factor name
V$GKLF_01	30 (+)	0.832	0.602	nanngnnnnANNGG	GKLF
V$GKLF_01	42 (+)	0.803	0.572	ggnncngnnGCNGG	GKLF
V$GKLF_01	63 (+)	0.784	0.586	gncagnnncNTNGG	GKLF
V$GKLF_01	64 (+)	0.888	0.638	ncagnnncnTNGGG	GKLF
V$GKLF_01	79 (+)	0.783	0.535	tcnnngnnnCNNGG	GKLF
V$GKLF_01	80 (+)	0.879	0.617	cnnngnnncNNGGG	GKLF
V$GKLF_01	81 (+)	0.92	0.613	nnngnnncnNGGGG	GKLF
V$P300_01	90 (+)	0.782	0.735	ngggGCNGTnnnan	p300
V$GKLF_01	101 (+)	0.788	0.576	nantnngncCCNGG	GKLF
V$SP1_01	109 (-)	0.855	0.808	cccNGGCCtg	Sp1
V$GKLF_01	109 (-)	0.89	0.759	CCCNGgcctgntgn	GKLF
V$GKLF_01	110 (-)	0.788	0.682	CCNGGcctgntgnc	GKLF
V$SP1_01	111 (+)	0.893	0.82	cnGGCCTgnt	Sp1
V$GKLF_01	115 (-)	0.852	0.618	CCTGNtgncnnnnn	GKLF
V$GKLF_01	135 (-)	0.879	0.642	CCCNNcnnncnnnn	GKLF
V$GKLF_01	136 (-)	0.798	0.535	CCNNCnnncnnnnn	GKLF

V$GKLF_01	176 (+)	0.832	0.59	annncnnnnNNAGG	GKLF
V$GKLF_01	186 (+)	0.798	0.641	naggngnnaGNNGG	GKLF
V$GKLF_01	187 (+)	0.879	0.756	aggngnnagNNGGG	GKLF
V$GKLF_01	221 (+)	0.879	0.586	cnnncnnnnNNGGG	GKLF
V$GKLF_01	222 (+)	0.92	0.595	nnncnnnnnNGGGG	GKLF
V$GKLF_01	223 (+)	0.946	0.62	nncnnnnnnGGGGG	GKLF
V$MZF1_02	226 (+)	0.805	0.712	nnnnnNGGGGgaa	MZF1
V$MZF1_02	227 (+)	0.85	0.706	nnnnnGGGGGaag	MZF1
V$GKLF_01	227 (+)	0.798	0.747	nnnnnggggGAAGG	GKLF
V$GKLF_01	228 (+)	0.879	0.826	nnnngggggAAGGG	GKLF
V$GKLF_01	229 (+)	0.92	0.83	nnngggggaAGGGG	GKLF
V$MZF1_01	230 (+)	0.843	0.969	nngGGGGA	MZF1
V$GC_01	231 (+)	0.808	0.822	ngggGGAAGgggnc	GC box
V$MZF1_02	233 (+)	0.805	0.782	ggggaAGGGGncn	MZF1
V$CDXA_01	233 (-)	0.758	0.717	ggGGAAG	CdxA
V$SP1_01	233 (+)	0.851	0.84	ggGGAAGggg	Sp1
V$GKLF_01	239 (+)	0.852	0.602	ggggncnnaNCNGG	GKLF
V$GKLF_01	240 (+)	0.89	0.678	gggncnnanCNGGG	GKLF
V$YY1_01	256 (-)	0.894	0.753	cnnnngngATGNGnncn	YY1
V$GKLF_01	262 (+)	0.783	0.614	ngatgngnnCNNGG	GKLF
V$GKLF_01	263 (+)	0.879	0.678	gatgngnncNNGGG	GKLF
V$GKLF_01	264 (+)	0.92	0.682	atgngnncnNGGGG	GKLF
V$GKLF_01	289 (+)	0.783	0.631	nngcnngagCNNGG	GKLF
V$GKLF_01	290 (+)	0.879	0.702	ngcnngagcNNGGG	GKLF
V$GKLF_01	291 (+)	0.92	0.72	gcnngagcnNGGGG	GKLF
V$GKLF_01	292 (+)	0.946	0.721	cnngagcnnGGGGG	GKLF
V$AP2_Q6	295 (-)	0.915	0.74	gagcnNGGGGgt	AP-2
V$AP2_Q6	296 (-)	0.992	0.853	agcnnGGGGGtg	AP-2
V$GKLF_01	297 (+)	0.821	0.73	gcnnggggqTGNGG	GKLF
V$P300_01	298 (+)	0.85	0.838	cnngGGGGTgnggc	p300
V$GC_01	300 (+)	0.798	0.844	ngggGGTGNggcng	GC box
V$SP1_01	302 (+)	0.806	0.82	ggGGTGNggc	Sp1
V$CEBP_01	302 (+)	0.839	0.65	ggGGTGNggcngn	C/EBP
V$PAX4_03	302 (-)	1	0.92	gGGGTGnggcng	Pax-4
V$CEBP_01	342 (-)	0.779	0.653	nccannGCANNna	C/EBP
V$GKLF_01	343 (-)	0.784	0.584	CCANNgcannnagn	GKLF
V$GKLF_01	347 (+)	0.832	0.671	ngcannnagNANGG	GKLF

V$GKLF_01	348 (+)	0.879	0.756	gcannnagnANGGG	GKLF
V$GKLF_01	359 (+)	0.803	0.699	gggcnanaaGCNGG	GKLF
V$GKLF_01	368 (+)	0.81	0.739	gcnggangaGNTGG	GKLF
V$CEBP_01	375 (+)	0.832	0.643	gaGNTGGnagnnn	C/EBP
V$GKLF_01	386 (+)	0.852	0.61	nnntgngnnNCAGG	GKLF

CR1-H subfamily   Graph version

Matrix identifier	Position (strand)	Core match	Matrix match	Sequence (always the (+)-strand is shown)	Factor name
V$CAP_01	89 (-)	0.983	0.97	gnnACTGN	cap
V$GR_Q6	101 (-)	0.98	0.851	nanGNACAgngngntttnn	GR
V$CDXA_01	114 (+)	0.882	0.805	NTTTNnn	CdxA
V$GKLF_01	134 (-)	0.784	0.598	CCANNgancccnga	GKLF
V$GKLF_01	142 (-)	0.89	0.652	CCCNGagntnanag	GKLF
V$GKLF_01	143 (-)	0.786	0.557	CCNGAgntnanaga	GKLF
V$CEBP_01	151 (-)	0.779	0.693	nanagaGCAGNcn	C/EBP
V$USF_02	162 (+)	0.869	0.603	cnngCAGCTctnnn	USF
V$AP4_Q6	164 (+)	1	0.862	ngCAGCTctn	AP-4
V$AP4_Q5	164 (+)	1	0.916	ngCAGCTctn	AP-4
V$CAP_01	165 (+)	0.978	0.974	GCAGCtct	cap
V$GKLF_01	274 (+)	0.888	0.571	nnnnnnnnnNGAGG	GKLF
V$GKLF_01	281 (+)	0.786	0.714	nnngaggagTCNGG	GKLF
V$P300_01	282 (+)	1	0.926	nngaGGAGTcnggn	p300
V$HSF2_01	283 (+)	0.848	0.867	NGAGGagtcn	HSF2
V$PAX2_01	285 (+)	0.776	0.544	aggaGTCNGgnnntgnnna	Pax-2
V$CEBP_01	294 (+)	0.767	0.732	gnNNTGNnnatcn	C/EBP
V$GKLF_01	298 (+)	0.784	0.566	tgnnnatcnNNTGG	GKLF
V$GKLF_01	299 (+)	0.891	0.653	gnnnatcnnNTGGG	GKLF
V$CEBP_01	311 (-)	0.885	0.689	ggnnntTCANAnn	C/EBP
V$CDXA_01	315 (+)	0.786	0.745	NTTCAna	CdxA
V$CDXA_01	350 (+)	0.786	0.716	NTTCAnn	CdxA
V$CEBP_01	394 (+)	0.822	0.832	nnNGTGNtaantn	C/EBP
V$EN1_01	397 (+)	0.913	0.879	GTGNTaa	En-1
V$GKLF_01	397 (+)	0.772	0.604	gtgntaantNNNGG	GKLF
V$GKLF_01	398 (+)	0.879	0.69	tgntaantnNNGGG	GKLF

V$MSX1_01	398 (+)	0.856	0.663	tgnTAANTn	Msx-1
V$CDXA_01	399 (-)	0.776	0.721	gnTAANT	CdxA
V$USF_02	409 (-)	0.935	0.685	gggnnACCTGttan	USF
V$CEBP_01	413 (+)	0.771	0.639	naCCTGTtannan	C/EBP
V$OCT1_03	413 (-)	0.905	0.853	nacctGTTANnan	Oct-01
V$GKLF_01	415 (-)	0.913	0.658	CCTGTtannannnc	GKLF
V$HOXA3_01	415 (+)	0.753	0.653	CCTGTtann	HOXA3
V$CDXA_01	418 (+)	0.772	0.732	GTTANna	CdxA
V$GKLF_01	426 (+)	0.772	0.574	nncctngtaNNNGG	GKLF
V$GKLF_01	428 (-)	0.859	0.611	CCTNGtannnggnn	GKLF
V$GKLF_01	433 (+)	0.772	0.616	tannnggnnNANGG	GKLF
V$GKLF_01	458 (+)	0.772	0.649	ctannnncaNNAGG	GKLF
V$PAX2_01	468 (-)	0.872	0.578	naggncngngCTGATnant	Pax-2
V$CAP_01	474 (-)	0.986	0.972	ngnGCTGA	cap
V$CEBP_01	475 (+)	0.792	0.805	gnGCTGAtnantc	C/EBP
V$PAX2_01	475 (-)	0.787	0.687	gngctgatnaNTCACannc	Pax-2
V$PBX1_01	478 (-)	0.819	0.728	cTGATNant	Pbx-1
V$MSX1_01	479 (+)	0.856	0.594	tgaTNANTc	Msx-1
V$HSF2_01	479 (+)	0.862	0.872	TGATNantca	HSF2
V$HSF2_01	479 (-)	0.837	0.876	tgatnANTCA	HSF2
V$AP1_Q6	480 (-)	0.896	0.877	gatnANTCAca	AP-1
V$CEBP_01	480 (-)	0.94	0.713	gatnanTCACAnn	C/EBP
V$PBX1_01	481 (+)	0.819	0.683	atnANTCAc	Pbx-1
V$PAX4_03	481 (+)	0.77	0.746	atnantCACANn	Pax-4
V$PAX2_01	481 (+)	0.831	0.591	atnaNTCACanncnattna	Pax-2
V$MSX1_01	483 (-)	0.857	0.611	nANTCAcan	Msx-1
V$CAP_01	486 (+)	0.946	0.962	TCACAnnc	cap
V$GKLF_01	488 (+)	0.856	0.698	acanncnatTNAGG	GKLF
V$MSX1_01	494 (-)	0.856	0.59	nATTNAggc	Msx-1
V$CDXA_01	494 (+)	0.776	0.717	NATTNag	CdxA
V$PAX4_04	507 (-)	0.879	0.604	nnnatagngctngtgagtctgctnnTTTGC	Pax-4
V$PAX2_01	509 (-)	0.84	0.564	natagngctnGTGAGtctg	Pax-2
V$USF_Q6	514 (-)	0.926	0.85	ngctNGTGAg	USF
V$PAX2_01	515 (+)	0.796	0.568	gctnGTGAGtctgctnntt	Pax-2
V$CEBP_01	516 (+)	0.822	0.638	ctNGTGAgtctgc	C/EBP
V$PAX4_03	516 (-)	0.77	0.781	cTNGTGagtctg	Pax-4
V$MSX1_01	517 (+)	0.865	0.658	tngTGAGTc	Msx-1

V$HAND1E47_01	517 (+)	0.871	0.825	tngtgagTCTGCtnnt	Hand1/E47
V$PAX2_01	519 (+)	0.822	0.536	gtgaGTCTGctnntttgcc	Pax-2
V$CAP_01	521 (-)	0.971	0.971	gagTCTGC	cap
V$CEBP_01	522 (+)	0.897	0.728	agTCTGCtnnttt	C/EBP
V$NF1_Q6	524 (-)	0.911	0.882	tctgctnntttGCCATng	NF-1
V$OCT1_Q6	528 (-)	1	0.68	ctnnTTTGCcatngc	Oct-01
V$OCT1_Q6	529 (-)	0.77	0.721	tnntTTGCCatngct	Oct-01
V$CEBP_01	530 (+)	0.972	0.81	nnTTTGCcatngc	C/EBP
V$CEBP_01	530 (+)	0.984	0.839	nnTTTGCcatngct	C/EBP
V$CEBP_01	530 (-)	0.806	0.811	nntttgCCATNgc	C/EBP
V$YY1_01	531 (+)	0.998	0.854	ntttGCCATngctnnnc	YY-1
V$CDXA_01	531 (+)	0.91	0.858	NTTTGcc	CdxA
V$CDXA_01	532 (+)	0.766	0.739	TTTGCca	CdxA
V$GKLF_01	536 (-)	0.784	0.653	CCATNgctnnnccn	GKLF
V$GKLF_01	557 (+)	0.859	0.568	nngntncnnCNAGG	GKLF
V$GKLF_01	567 (+)	0.784	0.758	naggaanaaNNTGG	GKLF
V$YY1_01	569 (-)	0.756	0.748	ggaanaanNTGGAgann	YY-1
V$CEBP_01	574 (+)	0.806	0.814	aaNNTGGagannn	C/EBP
V$CDXA_02	631 (-)	0.77	0.67	nAGAANa	CdxA
V$CEBP_01	644 (-)	0.885	0.665	nnaganNCANAnt	C/EBP
V$GKLF_01	647 (+)	0.821	0.69	ganncananTGNGG	GKLF
V$CEBP_01	652 (+)	0.767	0.677	anANTGNggtccn	C/EBP
V$AML1_01	656 (+)	0.928	0.937	tGNGGT	AML-1a

CR1-B subfamily Graph version

Matrix identifier	Position (strand)	Core match	Matrix match	Sequence (always the (+)-strand is shown)	Factor name
V$CEBP_01	99 (+)	0.759	0.74	nnCCTGNntagaa	C/EBP
V$GKLF_01	101 (-)	0.852	0.637	CCTGNntagaanaa	GKLF
V$CDXA_02	107 (-)	0.77	0.771	tAGAANa	CdxA
V$CDXA_01	117 (+)	0.765	0.757	CTTNGag	CdxA
V$CEBP_01	142 (-)	0.885	0.68	nnnncnTCAGAga	C/EBP
V$PAX2_01	143 (+)	0.801	0.564	nnncNTCAGaganggngag	Pax-2
V$GKLF_01	144 (+)	0.858	0.696	nncntcagaGANGG	GKLF
V$CAP_01	148 (+)	0.98	0.967	TCAGAgan	cap

V$MSX1_01	149 (-)	0.806	0.81	cAGAGAngg	Msx-1
V$GKLF_01	149 (+)	0.888	0.823	cagaganggNGAGG	GKLF
V$MZF1_01	156 (+)	0.852	0.846	ggnGAGGA	MZF1
V$CEBP_01	172 (+)	0.759	0.645	nnNNTGAnatcnn	C/EBP
V$CEBP_01	188 (+)	0.771	0.687	nnNCTGTtttaan	C/EBP
V$CEBP_Q2	189 (-)	0.773	0.764	nnctgttTTAANna	C/EBP
V$MSX1_01	189 (+)	0.818	0.572	nncTGTTTt	Msx-1
V$OCT1_03	190 (-)	0.916	0.85	nctgtTTTAAnna	Oct-01
V$CDXA_01	193 (+)	0.898	0.866	GTTTTaa	CdxA
V$EN1_01	193 (+)	0.922	0.887	GTTTTaa	En-1
V$OCT1_03	193 (+)	0.916	0.892	gttTTAANnannt	Oct-01
V$CDXA_01	194 (-)	0.839	0.811	ttTTAAN	CdxA
V$CDXA_02	194 (-)	0.856	0.806	tTTTAAn	CdxA
V$CDXA_02	194 (+)	0.856	0.742	tTTTAAn	CdxA
V$CDXA_01	194 (+)	0.968	0.901	TTTTAan	CdxA
V$CDXA_01	195 (+)	0.849	0.774	TTTAAnn	CdxA
V$CDXA_02	195 (+)	0.856	0.731	tTTAANn	CdxA
V$USF_02	197 (-)	0.826	0.593	taannANNTGnnac	USF
V$CAP_01	206 (-)	0.983	0.97	gnnACTGN	cap

CR1-F subfamily   Graph version

Matrix identifier	Position (strand)	Core match	Matrix match	Sequence (always the (+)-strand is shown)	Factor name
V$CEBP_01	1795 (+)	0.759	0.669	nnNNTGNtctcnn	C/EBP
V$GKLF_01	1923 (+)	0.772	0.554	nntncnnngNNNGG	GKLF
V$GKLF_01	1959 (+)	0.803	0.546	nnggnncnnGCNGG	GKLF
V$GKLF_01	1980 (+)	0.784	0.572	gnnncnnngNNTGG	GKLF
V$GKLF_01	1983 (+)	0.839	0.572	ncnnngnntGGNGG	GKLF
V$GKLF_01	2046 (+)	0.803	0.582	ngnntnngnGCNGG	GKLF
V$GKLF_01	2164 (-)	0.772	0.545	CCNNNnngtngnnn	GKLF
V$GKLF_01	2193 (+)	0.772	0.561	ncngnnnngNNNGG	GKLF
V$USF_02	2243 (+)	0.826	0.584	nnnnCANNTnccng	USF
V$GKLF_01	2253 (-)	0.777	0.603	CCNGNtcnnnnann	GKLF
V$GKLF_01	2257 (+)	0.772	0.565	ntcnnnnanNNNGG	GKLF
V$GKLF_01	2280 (-)	0.879	0.603	CCCNNnncnncnnn	GKLF

V$GKLF_01	2281 (-)	0.772	0.592	CCNNNncnncnnng	GKLF
V$GKLF_01	2333 (+)	0.772	0.586	gnnnnnggnNNNGG	GKLF
V$GKLF_01	2334 (+)	0.879	0.61	nnnnnggnnNNGGG	GKLF
V$GKLF_01	2335 (+)	0.92	0.651	nnnnggnnnNGGGG	GKLF
V$GKLF_01	2336 (+)	0.946	0.661	nnnggnnnnGGGGG	GKLF
V$CEBP_01	2364 (+)	0.759	0.751	anNNTGNnaanan	C/EBP
V$CEBP_01	2385 (+)	0.814	0.787	agNGTGNcnangn	C/EBP
V$PAX4_03	2385 (-)	0.876	0.787	aGNGTGncnang	Pax-4
V$GKLF_01	2395 (+)	0.798	0.65	ngnngnnnaGNAGG	GKLF
V$GKLF_01	2419 (-)	0.783	0.627	CCNNGcttnnnnnn	GKLF
V$SRY_01	2420 (-)	0.767	0.705	cnNGCTT	
V$GKLF_01	2445 (+)	0.772	0.543	cnannnnnnNNNGG	GKLF
V$GKLF_01	2554 (-)	0.95	0.629	CCCTGnngnnngnn	GKLF
V$GKLF_01	2555 (-)	0.777	0.551	CCTGNngnnngnnn	GKLF
V$GKLF_01	2610 (-)	0.772	0.579	CCNTNtgnnnnnnn	GKLF
V$CEBP_01	2611 (+)	0.877	0.65	cnTNTGNnnnnnn	C/EBP
V$GKLF_01	2677 (-)	0.772	0.665	CCTNNnngctntnn	GKLF
V$GKLF_01	2754 (+)	0.798	0.534	ntngnnnnnGNNGG	GKLF
V$GKLF_01	2776 (+)	0.777	0.57	cnnnnngngNCNGG	GKLF
V$GKLF_01	2777 (+)	0.95	0.654	nnnnngngnCNGGG	GKLF
V$GKLF_01	2835 (+)	0.789	0.545	ngntnngnnNCTGG	GKLF

Supplementary Table 5:
Nucleotide sequences of functionally active or inactive 5'UTR promoter of CR1-F subfamily

Functional 5'UTR of CR1-F
Chromosome 6
Position 2161469-2162179

```
CTGGAGAGCAAAGAGTTATCACTGGCATGCCAATTACAGTTAACAAGGAGAAGCTTG
GTCTGTTGGCAAGGCTACAGGGTGCCTTATGTTTTAATGACAACGGTGATCAAACAC
TGCATGAGTATGCTTCAGAATTAATATATTAATAGTGAGCAAAAGAGGTCCCTGTCC
ATGTGTAGGAGATGCCGTTACAGAAATAATGCAAATATTGATAGTAATTAGTCATGG
GGATCTCAGTGTAGTCTCTCTTTCTCTTGTATTCCTTCACAATGTCATGTCTTAAAA
GAATCGTTGGCTTCTATGGTTAATTTTTTGTACTTGGAAATTTTATTTTCATTATCC
TTTCATGCAGTGTTTTAGACCTTGGGATTATCAGGACTGCATTATGTCATGTATGCA
TCATGTACTTCAGAAACTTTTTAGCAGATCACTTCAGAAGCAGAAGTTGGGATATTA
GCAGGGAGGGGTCACGGCTATATTGTCATTCAAGAGACTGACAGGCACTTAGGCCAG
TTCTGATGAAGCGACCTAAACAAAGTGAGATGCTTTTGGAGGGCTGTAGATCTCTCA
TGTTCTCCCCCCATTAGTCCTGACTAATGTAGGAGCAAGTGGGTCTTGTGGCAGTAT
ACGTGAGTGTTTCTTTTGTTCCTTTCGTCTTCGCTTAGGAGACCAGCTCTCCTACGG
ACAGTCCCTGTGACCTCCAGTTTGAAC
```

Non-functional 5'UTR of CR1-F
Chromosome 1
Position 166387715-166388712

```
TGTCAGGTCTTGCATTACTCCATATATATCTGTAGGAAACTGAAATCATTACTCTGT
AGTGTTATGAACACTAAATTGAAAACAAAAACTTAATCCAAATCCCCATATTCTGGA
TTAAAACTAACTTTATTTTGAAAGCAAGTATTTTTCTTTGATGAATGCCTCTTTTTT
CTTTCTAGGTTACTTTGCAACTTGAAGGATACAGTAGCTCTCTACCCTGCTGAGTTT
TGAATTTCAGCTTTTAGTTGTTTTACTTTAATCTGAGGGAAAACTCCCATGTATTCA
AAAAGGTATAAGCCATTTAAAAAGTATCAACAAAAATATGCTTCCTCTCTGAACCTG
CATTCAGTATCCAACAATAAATTTGACACTGCAGCCACATTTTAGCCCCAGATCAGT
CACAATAAACATCAGTTGCAAACCTAGCAACTCAAATCAGCAAATTCAATATCACTT
GTCCTAGTTCTGCTAACCTTGCCGTGAACCTATCCATTTAGTCCAAGAGGTCCTAAC
CTTCATTGCTGCAGAGACCAGCTTCAGGGCAGGTAAGACTGCTCTGGACAAGGGACC
TAAAGCAAAAAGGCTGACCAAAAAGCTCTTTTTAGGGCTTTTTTGAAGCTAGCAAAA
GCACTATACCGGTGCCGGGGGGCTGGCAGCTTGTGGGCGGGCTACTGAGCCAGCAGG
GGCAGAGCTGGCACTGCCCACTCTTATCTCGCGAGCTCGACCTTGACCTTTTGCTGT
GTGACTAGAAGCATCTTTGATAGAGTGGAAGCTAGCTTCCACCCCCTTAACTCACTA
GATGAGAAATGGGTGTTACCTTCCACCGTTAACAGGAAGGGAGTCAGTGTGTGAGCG
CAGCCCACGCTGCCCATACTGCAGCAGCAGGTGTGGGAGGTGGGTCTTGTGGCAGGA
TACGTGAGTGTTTCTTTTGTTCCTTTTGTCTTCACTTAGGAGACAAGCTCTCCTACA
CAGTCCCTGTGACCTGCAATTTGAAC
```

Supplementary Table 6: Full-length CR1-F and CR1-B elements in the chicken genome

Subfamily	Strand	Chromosome	Position	Length	Activity
CR1-F	+	Chr.6	2161469-2166909	5440 bp	Functional element with active 5'UTR promoter with intact ORF1 and ORF2.
CR1-F	+	Chr.7	9136370-9140059	3689 bp	Non-functional due to truncation at 5'UTR promoter and ORF1; contains an intact ORF2 coding region. GenBank Acc. AF308605
CR1-F	_	Chr.1	35365502-35361782	3720 bp	Non-functional due to mutation at initiation codon and ORF1 region; GenBank Acc. Af308604
CR1-F	+	Chr.6	2162080-2166122	4042 bp	Non-functional due to frame-shift mutation at ORF1 and ORF2 regions; GenBank Acc. AF308540
CR1-B	_	Chr.2	51121971-51126431	4461 bp	Potentially active element due to presence of promoter-like sequences; contains only ORF2 coding region
CR1-B	+	Chr.1	9703052-9707292	4241 bp	Non-functional due to absence of 5'UTR promoter; contains intact ORF1 and ORF2.
CR1-B	_	Chr.5	5715277-5719735	4459 bp	Functional element with active 5'UTR promoter with both ORF1 and ORF2. GenBank Acc.U88211
CR1-B	_	Chr.5	4052844-4057054	4211 bp	Non-functional due to absence of 5'UTR promoter; contains intact ORF1 and ORF2.
CR1-B	_	Chr.2	57235637-57239713	4077 bp	Non-functional due to absence of 5'UTR promoter; contains intact ORF1 and ORF2.
